# Anti-Inflammatory Effects of Cicadidae Periostracum Extract and Oleic Acid through Inhibiting Inflammatory Chemokines Using PCR Arrays in LPS-Induced Lung inflammation In Vitro

**DOI:** 10.3390/life12060857

**Published:** 2022-06-08

**Authors:** Jung-Hee Hong, Young-Cheol Lee

**Affiliations:** Department of Herbology, College of Korean Medicine, Sangji University, 83, Sangjidae-gil, Wonju-si 26339, Korea; anifam2030@sangji.ac.kr

**Keywords:** chemokine gene profiling, chemokines, chemokine receptors, Cicadidae Periostracum ethanol extract, oleic acid, allergic asthma, LPS, lung inflammation, mouse lung epithelial cells, mouse lung macrophages

## Abstract

In this study, we aimed to evaluate the anti-inflammatory effects and mechanisms of CP and OA treatments in LPS-stimulated lung epithelial cells on overall chemokines and their receptors using PCR arrays. In addition, we aimed to confirm those effects and mechanisms in LPS-stimulated lung macrophages on some chemokines and cytokines. In our study, CP treatments significantly inhibited the inflammatory mediators CCL2, CCL3, CCL4, CCL5, CCL6, CCL9, CCL11, CCL17, CCL20, CXCL1, CXCL2, CXCL3, CXCL5, CXCL7, CXCL10, TNF-α, and IL-6, while markedly suppressing NF-κB p65 nuclear translocation and the phosphorylations of PI3K p55, Akt, Erk1/2, p38, and NF-κB p65 in LPS-stimulated lung epithelial cells. CP treatments also significantly decreased the inflammatory mediators CCL2, CCL5, CCL17, CXCL1, and CXCL2, while markedly inhibiting phospho-PI3K p55 and iNOS expression in LPS-stimulated lung macrophages. Likewise, OA treatments significantly suppressed the inflammatory mediators CCL2, CCL3, CCL4, CCL5, CCL8, CCL11, CXCL1, CXCL3, CXCL5, CXCL7, CXCL10, CCRL2, TNF-α, and IL-6, while markedly reducing the phosphorylations of PI3K p85, PI3K p55, p38, JNK, and NF-κB p65 in LPS-stimulated lung epithelial cells. Finally, OA treatments significantly inhibited the inflammatory mediators CCL2, CCL5, CCL17, CXCL1, CXCL2, TNF-α, and IL-6, while markedly suppressing phospho-PI3K p55, iNOS, and Cox-2 in LPS-stimulated lung macrophages. These results prove that CP and OA treatments have anti-inflammatory effects on the inflammatory chemokines and cytokines by inhibiting pro-inflammatory mediators, including PI3K, Akt, MAPKs, NF-κB, iNOS, and Cox-2. These findings suggest that CP and OA are potential chemokine-based therapeutic substances for treating the lung and airway inflammation seen in allergic disorders.

## 1. Introduction

Cicadidae Periostracum (CP) is a crude drug derived from the cast-off skin of the insect Cryptotympana pustulata Fabricius from the family of Cicadas [1,2]. CP has been used to treat smallpox, sedation, shock, edema, epilepsy, and night terror symptoms in traditional Korean medicine. Various pharmacological studies on CP have shown its diverse biological properties, such as neuroprotective [3]; immunomodulatory [4]; anti-fibrosis [1]; hypothermic, sedative, and anti-convulsive [5]; anti-oxidative [6]; and anti-inflammatory [2,4,6] effects. CP is predominantly composed of proteins and chitin and a small number of minerals, lipids, and other constituents [4,7,8]. Among the numerous components that make up a small proportion of CP, oleic acid (OA) accounts for a relatively large proportion of the total fatty acids in the ethanol extract of CP, which was previously reported by our group [7]. OA is a monounsaturated and non-essential fatty acid and has demonstrated anti-inflammatory [9,10,11,12] and anti-tumor [13] activities. Although the role of OA in immune responses is still controversial [9,10], previously, our group demonstrated the anti-asthmatic effects of CP and OA in OVA-induced asthmatic mouse models [7].

Allergic asthma is a chronic airway disease that involves complex interactions of genetic and environmental factors to influence asthma endotypes or biological processes [14,15]. The main types of the latest classification of allergic respiratory diseases focus on the characterization of the endotype based on distinct functional or pathophysiological mechanisms and the phenotype based on the description of the condition [15,16]. Asthma is characterized by symptoms such as wheezing, chest tightness, shortness of breath, and cough. These symptoms arise due to chronic airway inflammation in response to triggers, commonly inhaled allergens, which lead to airway hyperresponsiveness (AHR), causing variable airway narrowing, airway remodeling, and mucus hypersecretion. The complexity of variation in the symptoms and its pathogenesis make asthma a more complicated heterogeneous syndrome with some endotypes [17]. The development of chronic airway inflammation due to inappropriate airway immune responses to specific pathogens or allergens has been identified as a crucial pathological feature of asthmatics [18]. Classical Th2 cell-mediated eosinophilic inflammation is associated with a subset of patients with severe asthma and is primarily identified by IgE levels and blood eosinophil counts in clinical evaluations [15,19]. Th2 and non-Th2 are the main endotypes of respiratory allergies. Recently, mixed endotypes such as Th2/Th17 or Th1/Th17 have been proposed [16]. Th2 cells orchestrate allergic inflammation by releasing Th2 cytokines IL-4, IL-5, and IL-13 [20,21]. Th2 cytokines are responsible for eosinophil accumulation and activation, IgE production, and airway abnormalities, including mucus metaplasia, bronchial hyperresponsiveness, and airway wall thickening [22]. Our study employed the two cell types—lung epithelial cells and lung macrophages—ultimately responsible for the airway inflammation in asthma pathogenesis.

Alveolar epithelial cells (AECs) are composed of type Ⅰ and type Ⅱ cells [23]. Type Ⅰ AECs form a barrier that is able to sense microbial products, generate inflammatory responses, and participate in gas exchange. Type Ⅱ AECs protect the lungs by secreting antimicrobial molecules such as lysozyme, complement, and surfactant proteins [24,25]. Type Ⅱ AECs release various cytokines and chemokines involved in the migration, activation, and differentiation of immune cells. Murine and human type Ⅱ AECs express major histocompatibility complex (MHC) class Ⅱ molecules and can present antigens to CD4+ T cells [26]. Evidence suggests that asthma is primarily an epithelial disorder. An altered epithelial barrier function exposes the airways, which are fragile in genetically susceptible individuals, to early life virus infections, which stimulate immature dendritic cells and induce local allergen sensitization. If the repair responses of the epithelial barrier are inadequate and there is sustained epithelial susceptibility to allergens, they lead to the persistence of asthma and produce various mediators, leading to airway wall remodeling and prolonged inflammation, which damage the barrier [27]. Maintaining the structural and functional homeostasis of airway epithelial cells is just as crucial as suppressing excessive inflammation. Once the defense of mucociliary clearance is breached, the airway epithelial cells contact pathogens that infect the respiratory tract [14,17]. The airway epithelial cells rapidly respond to microbes, cellular stress, or tissue damage by recognizing pathogen-associated molecular patterns (PAMPs) through membrane-bound or cytoplasmic pattern recognition receptors (PRRs) and initiate appropriate signaling before immune cells are recruited to the infection site. Epithelial PRR activation induces the production of chemokines and cytokines, playing an essential role in initiating allergic responses, which lead to the release of chronic and high levels of pro-inflammatory mediators that can mediate the lung pathology seen in asthma [18,27,28].

Lung macrophages are the most abundant innate immune cells and participate in respiratory defense, displaying a high phagocytic capacity and antigen presentation [25,29]. In particular, alveolar macrophages (AMs) participate in pro- and anti-inflammatory functions in allergic asthma [30]. In both asthmatics and murine models of allergic asthma, after allergen challenge, the rapid recruitment of monocytes occurred and there was an increase in the monocyte-derived population of AMs that promoted acute allergic lung inflammation [31]. After repeated exposure to allergens, inflammation becomes chronic due to the expanded recruitment of immune cells and consequently elevated levels of cytokines, leading to macrophage polarization [22]. Significantly, plastic macrophages integrate signals from their microenvironment, leading to context-dependent polarization into M1/classically orM2/alternatively activated macrophages, which represent the two extremes of a broad spectrum of divergent phenotypes [32]. The polarization is defined mainly by in vitro studies, and tissue macrophages are possibly activated along a continuum between M1 and M2 by variously combined stimuli in vivo [33]. Therefore, M1 and M2 constitute an inadequate approach for describing the scope of macrophage populations in vivo [22]. The regulating process of macrophage polarization is a complex interaction among various signaling molecules, including cytokines and chemokines [33]. The M1 phenotype can be induced by potent stimulants, such as lipopolysaccharide (LPS) and IFN-γ. The M2a subtype of M2 phenotype could be induced by IL-4 or IL-13 [34,35]. In general, M1 macrophages are associated with neutrophilic inflammation, and they secrete a quantity of CCL2 and CCL5 chemokines and Th1 cytokines [33]. On the other hand, M2a cells secrete a quantity of IL-13, CCL17, CCL18, CCL22, and CCL24, which activate Th2 cells and eosinophil inflammation in the lungs [22,33]. Unbalanced activation of macrophages is associated with several diseases. In asthma, one or both of these phenotypes are constantly activated, causing chronic inflammation and further damage to the airways [22,32]. Previous studies reported distinct macrophage phenotypes in allergic (house dust mite (HDM)-induced) and nonallergic (farm dust extract (FDE)-induced) lung inflammation of murine models. M1 macrophages were more prevalent in the FDE-induced nonallergic model and induced Th1/Th17 lung inflammation associated with neutrophil infiltration and severe asthma with poor corticosteroid response. Meanwhile, M2 macrophages predominated in the lungs of HDM-induced allergic inflammation, which is usually related to Th2 reactions with high levels of serum IgE, significant numbers of eosinophils, and Th2 cytokines. In both models, an increased number of lung macrophages was a common feature [36,37].

Chemokines are one of the earliest reactions in the homeostatic and inflammatory responses. The inflammatory reaction is a defensive mechanism to localize and eliminate harmful substances and components of impaired tissue [38]. Chemokines, signaling proteins that indicate the direction for cell migration, are a large family of small cytokines [38,39]. It is a prominent feature of chemokines to recruit leukocytes, and those recruited cells produce other chemokines that contribute to the next wave of leukocyte homing [39,40]. The other feature is the complex interactions between chemokines and their receptors [38]. According to their behavior characteristics, the four main subfamilies (CXC, CC, CX3C, and C) can also be divided into three groups, inflammatory, homeostatic, and dual-type chemokines [41,42,43,44]. Inflammatory chemokines, such as CCL2, 3, and 4, are induced in leukocytes and other cells in peripheral tissues in response to various threats to the organism, including microbial pathogens, irritants, or toxic cellular components [41,44]. They can also be expressed by pro-inflammatory stimuli, such as IL-1, TNF-α, and LPS [44]. On the other hand, homeostatic chemokines, such as CCL18, 27, and CXCL14, are constitutively expressed by resting cells, mediating the migration and positions of leukocytes in a steady state. The dual-type chemokines, such as CCL20 and 22 and CXCL9 and 10, cover both functions depending on different organs, tissues, or disease conditions. However, these classifications are not fixed and work fluidly [41,44]. Chemokine receptors are divided into two groups based on their mechanisms of action: G protein-coupled receptors (GPCRs) activating signals through G proteins, and atypical chemokine receptors (ACKRs) acting by binding with β-arrestin [45,46].

Although chemokines play essential roles in inflammatory responses, few studies have been performed on the anti-inflammatory effects and mechanisms of CP and OA on chemokine gene expression profiling. Functional analysis of the overall profile of chemokines and their receptors is more important than the analysis of specific chemokines. We hypothesized that CP and OA may demonstrate their anti-inflammatory effects by modulating inflammatory chemokines and their receptors in the lung inflammation shown in asthma. Therefore, in this study, we aimed to assess the anti-inflammatory effects and mechanisms of CP ethanol extract and OA on chemokines and their receptors’ gene expression utilizing LPS-stimulated mouse lung epithelial cells and lung macrophages in vitro. In particular, we conducted comprehensive gene expression profiling on the chemokines and their receptors in mouse lung epithelial cells. We tried to characterize the classical Th2 and non-Th2 endotypes with chemokines discriminately from the classification using interleukins.

## 2. Materials and Methods

### 2.1. Chemicals and Reagents

Oleic acid (Cat. no. O1383), lipopolysaccharides (LPS, Cat. no. L5418), and dimethyl sulfoxide (DMSO, Cat. no. D2650) were purchased from Sigma Chemical Co. (St. Louis, MO, USA). RPMI 1640 Medium with HEPES, RPMI 1640 Medium, antibiotic-antimycotic (100×), and SYBR Green PCR MasterMix were purchased from Thermo Fisher Scientific (Waltham, MA, USA). Fetal bovine serum (FBS) was purchased from Welgene (Gyeongsan-si, Korea). An RNeasy^®^ Mini Kit (Cat. no. 74104), RT2 First Strand kit (Cat. no. 330401), RT2 Profiler PCR Arrays (Cat. no. RAMM-022ZC), and RT2 SYBR Green/RoxqPCRMastermix (Cat. no. 330524) were purchased from Qiagen Sciences Inc. (Germantown, MD, USA). An EZ-Cytox cell viability assay kit was purchased from Daeilab Service co., Ltd. (Seoul, Korea). Ambion TRIzol^®^ reagent was purchased from life technologies (Grand Island, NY, USA). ReverTraAce cDNA synthesis kit was purchased from Toyobo Co., Ltd. (Osaka, Japan). The other chemicals were all analytical or cell culture grade and were purchased from Sigma Chemical Co. (St. Louis, MO, USA). Primary antibodies against iNOS, Cox2, Phospho-Erk1/2 (Thr202/Tyr204), Erk1/2, Phospho-p38 MAPK (Thr180/Tyr182), p38 MAPK, Phospho-SAPK/JNK (Thr183/Tyr185), SAPK/JNK, Phospho-NF-κB p65 (Ser536), NF-κB p65, Phospho-Akt (Ser473), Akt, Phospho-PI3K p85 (Tyr458)/p55 (Tyr199), PI3K p85, PI3K p55, β-actin, Vinculin, Cofilin, GAPDH, C23, and horseradish peroxidase-conjugated secondary antibodies were purchased from Cell Signaling Technology, Inc. (Danvers, MA, USA). ELISA kits were purchased from R&D Systems, Inc. (Minneapolis, MN, USA). Oligonucleotide primers were synthesized from Genotech Corporation (Daejeon, Korea).

### 2.2. Test Sample Preparation

Cicadidae Periostracum (CP) was purchased from Jeil Pharmaceutical Co. (Wonju, Korea). The dried and chopped CP was extracted 3 times with 70% ethanol using a 3 h reflux. The materials were filtered under reduced pressure in a vacuum rotary evaporator (BUCHI B-480, Buchi, Flawil, Switzerland) at 40 °C. Then, they were dried to yield the CP extract in a freeze-dryer (EYELA FDU-540, Tokyo, Japan), and the extract was stored at 4 °C. The yield of the extract from the initial crude material was roughly 5%. The CP extract and OA were each dissolved in DMSO for the experiment, and the final concentration of DMSO did not exceed 0.2%. All stocks were stored at –20 °C.

### 2.3. Cell Culture and Sample Treatment

We obtained the mouse lung epithelial cell line (LA-4) and mouse alveolar macrophage cell line (MH-S) from the Korean Cell Line Bank (Seoul, Korea). LA-4 cells were maintained in complete RPMI-1640 with HEPES supplemented with 10% FBS, 100 units/mL of penicillin, 100 μg/mL of streptomycin, and 0.25 μg/mL of amphotericin B in a humidified incubator (37 °C and 5% CO_2_). MH-S cells were maintained in complete RPMI-1640 supplemented with 10% FBS, 0.05 mM of β-mercaptoethanol, 100 units/mL of penicillin, 100 μg/mL of streptomycin, and 0.25 μg/mL of amphotericin B in a humidified incubator at 37 °C and 5% CO_2_. Before treatment, cells were plated at 5 × 10^5^ cells/mL density and incubated overnight. Both cell lines were stimulated with LPS at a concentration of 0.5 μg/mL for 1 h, followed by CP or OA treatments at the indicated concentrations, and incubated at 37 °C in a 5% CO_2_ incubator for 6 h or 24 h for RNA samples or protein samples, respectively.

### 2.4. Water Soluble Tetrazolium Salt (WST) Cell Viability

Cell viability was analyzed using the water-soluble tetrazolium (WST) assay. Cells were seeded at a density of 1 × 10^4^ cells/well in 96-well culture plates overnight. Then, they were exposed to various concentrations of the test compounds with or without LPS. Cells were incubated for 24 h at 37 °C in a 5% CO_2_ incubator, and during the last 4 h, WST (Ez-cytox) solution was added to each well according to the manufacturer’s instructions. Then, absorbance was read by a microplate reader (Epoch micro-volume spectrophotometer system, BioTek Inc., Santa Clara, CA, USA) at 450 nm and 600 nm as a reference. The background was adjusted by measuring the absorbance of cell-free medium containing WST solution.

### 2.5. RNA Extraction and Gene Expression Profiling Analysis

Total RNA was isolated using the RNeasy^®^ Mini kit. Then, the RNA was reverse transcribed into template cDNA from 0.5 μg total RNA of each sample for a 96-well plate (an array) using the RT^2^ first strand kit according to the manufacturer’s instructions. Briefly, cells were first lysed in buffer RLT, and the lysate was mixed with ethanol and then centrifuged using an RNeasy Mini spin column to bind the RNA to the silica membrane in the column. Traces of DNA that might co-purify with the RNA were removed by DNase treatment on the silica membrane. DNase and any contaminants were efficiently washed away, and high-quality total RNA was eluted in RNase-free water. Total RNA was quantified using a microplate reader (Epoch micro-volume spectrophotometer system, BioTek Inc., Santa Clara, CA, USA). Finally, the transcribed cDNA was ready for use in the downstream application according to the following two steps: elimination of genomic cDNA contamination and reverse transcription. The template cDNAs were characterized in technical triplicate using the Mouse Chemokine and Receptors RT^2^ Profiler PCR Arrays with RT^2^ SYBR Green/Rox qPCR Mastermix on the StepOnePlus Real-Time PCR system (Applied Biosystems, Foster City, CA, USA) according to the manufacturer’s instructions. The RT^2^ Profiler PCR Array plate contains the predispensed gene-specific primer sets for 84 genes that encode mouse chemokines and their receptors, as well as other related genes. In addition, each array includes five housekeeping genes and three RNA quality control elements for monitoring: genomic DNA contamination control (GDC), reverse transcription controls (RTC), and real-time PCR efficiency control (PPC). According to the manufacturer’s protocol, RNA quality was determined using RT^2^ RNA QC PCR arrays. The following PCR conditions were used: hold for 10 min at 95 °C and 60 s at 60 °C. The threshold was manually defined using the log view of the amplification plots, and the threshold cycle (CT) cut-off was set to 35. qPCR was performed in triplicate for each gene, and the quality was controlled by verifying a single peak in the melting curve analysis.

The CT values of target genes were normalized to an average of two housekeeping genes, GAPDH and ACTB. The comparative CT method was then used to examine the relative quantification of the samples. Fold changes in gene expression were then calculated for pairwise comparison using the equation 2-ΔΔCT. Fold change values greater than 1 indicate a positive or up-regulation, and fold change values less than 1 indicate a negative or down-regulation. The fold regulation is the negative inverse of the fold change. Fold regulation represents fold-change results in a biologically meaningful way. For example, a fold regulation value of −4.0 occurred when the normalized expression of a target gene in a test group was 4 times lower than that in the control group. The fold regulation threshold cut-off was set to at least 1.5.

### 2.6. Reverse Transcription–Quantitative Polymerase Chain Reaction (RT-qPCR) Analysis

The total RNA of LA4 and MH-S were extracted using Ambion TRIzol^®^ reagent and reverse-transcribed into cDNA using a ReverTraAce cDNA synthesis kit according to the manufacturer’s protocol. Total RNA was quantified using a microplate reader (Epoch micro-volume spectrophotometer system, BioTek Inc., Santa Clara, CA, USA). A total of 100 ng of cDNA and 10 pmole of gene-specific forward and reverse primers were loaded in the qPCR system (StepOnePlus Real-Time PCR system, Applied Biosystems, Foster City, CA, USA). Different types of chemokines and cytokines were amplified with SYBR Green PCR MasterMix. Mouse primer sequences for qRT-PCR are listed in Table 1. The oligonucleotides were manufactured by Genotech Corporation (Daejeon, Korea). The following amplification protocol was used: 2 min at 50 °C, 2 min at 95 °C, then 40 cycles of 15 sec at 95 °C and 1 min at 60 °C. The CT values of target genes were normalized to that of GAPDH. For each gene, qPCR was performed in triplicate, and the quality was controlled by verifying a single peak in the melting curve analysis. The comparative CT method was then used to examine the relative quantification of the samples.

### 2.7. Enzyme-Linked Immunosorbent Assay (ELISA)

The concentrations of different chemokines and cytokines in cell culture supernatants were determined by enzyme-linked immunosorbent assay (ELISA) according to the manufacturer’s instructions using antibody pairs from R&D Systems, Inc. (Minneapolis, MN, USA). Cell culture supernatants were collected, and the debris was removed by centrifugation at 1000× *g* for 10 min at 4 °C, followed by –80 °C freezing before analysis. The optical density of each well was immediately determined using a microplate reader (Epoch micro-volume spectrophotometer system, BioTek Inc., Santa Clara, CA, USA) at the recommended wavelength of the manufacturer.

### 2.8. Western Blot Analysis

Cells were lysed with RIPA lysis buffer (Sigma Chemical Co., St. Louis, MO, USA) for whole lysates, and the cell debris was removed via micro-centrifugation at 8000× *g* for 10 min at 4 °C. For cell fractionation, cytoplasmic and nuclear extracts were separated using the Nuclear/Cytosol Fractionation Kit (Biovision Incorporated, Milpitas, CA, USA) according to the manufacturer’s instructions. The protein concentration was determined by the Bradford method using Bio-Rad protein assay reagent (Bio-Rad Laboratories, Inc., Hercules, CA, USA) according to the manufacturer’s protocol. Total protein was diluted with sample buffer stock solutions (Laemmli’s SDS-sample buffer) to a final sample buffer concentration of 1× and incubated at 100 °C for 10 min. The prepared protein samples were subjected to various quantities (4–20%) of sodium dodecyl sulfate–polyacrylamide gel electrophoresis (SDS-PAGE) gel depending on the size of the protein of interest. Electrophoresed proteins were transferred onto nitrocellulose membranes using an electrophoretic transfer system (Bio-Rad Laboratories, Inc., Hercules, CA, USA). Then, the membranes were blocked with 5% skim milk in Tris-buffered saline (50 mM Tris-HCL, 150 mM NaCl, pH 7.5) with 0.1% Tween 20 for 1 h at room temperature. The membranes were then incubated overnight with each specific primary antibody at 4 °C. After washing, the membranes were incubated with each specific horseradish peroxidase (HRP)-conjugated secondary antibody for 2 h at room temperature. Finally, the blots were developed via enhanced chemiluminescence detection reagents (GenDEPOT, Katy, TX, USA). The densities of the bands on immunoblots were measured by Image J (available from the National Institutes of Health).

### 2.9. Statistical Analysis

For the RT^2^ Profiler PCR Array, *p*-values were calculated based on a Student’s *t-*test of triplicate 2^ (-Delta CT) values for each gene between 2 comparison groups, and only *p*-values less than 0.05 were included in the data. The data are presented as the mean ± SEM (standard error of the mean) of triplicate experiments for the other experiments. Statistically, significant differences were assessed by one-way analysis of variance (ANOVA), followed by Student’s *t-*test using SPSS statistical analysis software (version 14.0, IBM, Armonk, NY, USA). Statistical significance is indicated in the figures and tables.

## 3. Results

### 3.1. Chemokines and Their Receptors’ Gene Expression Profiles in Lung Epithelial Cells

#### 3.1.1. The Gene Expression Profiles of Chemokines and Their Receptors in LPS Stimulated Lung Epithelial Cells Using Mouse Chemokines and Receptors RT^2^ Profiler PCR Arrays

Mouse Chemokines and Receptors RT^2^ Profiler PCR arrays were used to explore the global gene expression patterns in LPS-stimulated LA4 cells. The array plate contains the predispensed gene-specific primer sets for 84 genes that encode numbers of C-C and C-X-C motif subfamilies of mouse chemokines and their receptors, as well as other related genes. The list of 84 genes is available on the manufacturer’s website. The RNAs for this assay were qualified by RT^2^ RNA quality control PCR Array (data not shown), which contains five housekeeping genes and three RNA quality control elements to monitor genomic DNA contamination, reverse transcription, and real-time PCR efficiency. These factors can lead to unreliable results in SYBR real-time PCR. By considering the LPS-induced genes and the genes that were significantly changed by CP or OA treatments based on our preliminary experiments (data not shown), 27 out of the 84 genes were selected. Then, custom RT^2^ Profiler PCR arrays were prepared. For the following experiments, the custom RT^2^ Profiler PCR arrays were used, unless otherwise noted, to confirm the efficacy of CP and OA treatments on chemokines and their receptors’ gene expression in LPS-stimulated LA4 cells. Those 27 genes are listed in Table 2. Over- and under-expressed genes in the LPS-stimulated group compared with the normal group are shown in Table 3. The mRNA gene expression level of CXCL12 was significantly decreased by LPS stimulation. The mRNA expression levels of chemokines CCL20, CXCL5, CXCL1, CCL2, CXCL10, CXCL11, CCL7, IL-6, CXCL3, CCL17 CXCL2, CCL8, CCRL2, TLR2, PPBP (CXCL7), CCL4, CCL5, and CCL9 were significantly increased in the LPS-stimulated group compared with the normal group.

Interestingly, CXCL12 expression was reduced considerably by LPS stimulation and it did not recover again following CP or OA treatments. CXCL12 is a dual-type chemokine. The function of homeostatic chemokines can be disrupted under inflammatory conditions, but it is not necessarily blocked [41]. A more detailed explanation of the reduced expression of CXCL12 is covered in the Discussion section.

#### 3.1.2. The Gene Expression Profiles of Chemokines and Their Receptors by CP Treatments in LPS-Stimulated LA4 Cells Using the Custom Mouse Chemokines and Receptors RT^2^ Profiler PCR Arrays

To investigate the effects of CP treatments on the mRNA gene expression of chemokines and their receptors in LPS-stimulated LA4 cells, qRT-PCR was performed using custom RT^2^ Profiler PCR arrays. Over- and under-expressed genes in the LPS-stimulated and CP 200 μg/mL-treated group compared with the LPS-stimulated group are listed in Table 4. Over- and under-expressed genes in the LPS-stimulated and CP 100 μg/mL-treated group compared with the LPS-stimulated group are listed in Table 5. Although there was a slight difference in the order of the width of change caused by CP treatments, the mRNA genes that were decreased in both groups were the same type, and none of the genes were increased compared with the LPS-stimulated group. The mRNA gene expression levels of chemokines CCL4, CCL9, CXCL3, CCL17, CXCL2, CCL6, CXCL10, and CCL20 were significantly down-regulated in the same manner in both CP 200 μg/mL-treated and CP 100 μg/mL-treated groups compared with the LPS-stimulated group. The PPBP (CXCL7) chemokine gene expression level was markedly down-regulated in the 200 μg/mL CP-treated group compared with the LPS-stimulated group.

As inflammatory chemokines, CCL4, CXCL2, CXCL3, CXCL7, and CXCL10 can be upregulated under inflammatory conditions. CCL4, known as macrophage inflammatory protein-1β (MIP-1β), is produced by neutrophils, monocytes, B cells, T cells, fibroblasts, endothelial cells, and epithelial cells. CCL4 binding to its receptors, CCR5 and CCR8, initiates the migration of natural killer cells, monocytes, and T cells to the inflamed area and induces the activation of granulocytes and T cells, T cell differentiation, and dendritic cell maturation [47]. Menten et al. investigated the inflammatory activities of CCL3 and CCL4 both in vitro and in vivo [48]. Capelli et al. observed a significant difference in CCL4 level in bronchoalveolar lavage fluid (BALF) and demonstrated its roles in the pathogenesis of chronic bronchitis [49]. CXCL2, CXCL3, and CXCL7 were found to be associated with the function of Th17 lymphocytes. The primary role of these chemokines is to attract and activate neutrophils via their receptor CXCR2. Essential evidence that these chemokines contribute to the allergic inflammation of respiratory diseases was obtained through experimental murine models [16].

CXCL10 is an inflammatory chemokine produced during an inflammatory response via the activation of resident cells and leukocytes. CXCL10 acts on immune cells expressing CXCR3 and CXCR4, such as eosinophils, neutrophils, monocytes, and T lymphocytes, particularly Th1 cells via CXCR3. CXCL10 was proven to be a useful inflammatory marker of occupational asthma exacerbation caused by wood dust [16].

CCL17 promotes the recruitment, activation, and development of Th2-polarized cells expressing CCR4 and plays an essential role in the development of lung diseases. TNF-α, in combination with IL-4 or IL-13, upregulates the CCL17 expression in epithelial cells. As for CCL20, the secretion of CCL20 is substantially increased by pathogenic substances in the pathogenesis of asthma. CCL20 recruits monocytes or immature DCs to the site of inflammation and promotes their maturation via its receptor, CCR6, and then DCs move to the mediastinal lymph nodes and activate T cells [18].

There are few studies on the roles of CCL6 and CCL9 in asthma. Nevertheless, the functions of CCL6 and CCL9 in relation to DCs’ migration and lung inflammation in mice have been shown. Despite this, they have only been identified in rodents. CCR1 is their common receptor, and CCR3 is the other receptor of CCL9. Macrophages express CCL6 and CCL9 and their receptor, CCR1. It was reported that CCL9 is secreted from the follicle-associated epithelium of mouse Peyer’s patches, where CD11b+ DCs expressing CCR1 migrated toward CCL9 [50]. Moreover, increased mRNA expression of CCL9 was found in the lung tissue of LPS-inoculated mice [51]. Huang et al. demonstrated the promoted expression of the inflammatory chemokine CCL6 in alveolar epithelial cells induced by IL-31, which is involved in lung inflammation. The supernatant of IL-31-stimulated alveolar epithelial cell culture enhanced the migration of macrophages and T cells in vitro [52].

Our results demonstrated that CP treatments showed specific inhibitory effects on inflammatory chemokines CCL4, CCL9, CXCL3, CCL17, CXCL2, CCL6, CXCL10, CCL20, and CXCL7. CP treatments could exhibit anti-inflammatory effects by controlling the recruitment of dendritic cells, monocytes, macrophages, eosinophils, neutrophils, and T cells and by modulating T cell differentiation, dendritic cell maturation, and Th1/Th2/Th17 lymphocyte functions based on the roles of the chemokines reversed by CP treatments.

#### 3.1.3. The Gene Expression Profiles of Chemokines and Their Receptors by OA Treatments in LPS-Stimulated LA4 Cells Using the Custom Mouse Chemokines and Receptors RT^2^ Profiler PCR Array

Previously, our group reported that OA accounts for a relatively large proportion of total fatty acids in the ethanol extract of CP [7]. The data from our previous study are shown in Table S1 and Figure S1. The roles of OA in immune responses are still controversial, as mentioned earlier [9,10]. Therefore, to explore the effects of OA treatments on the mRNA gene expression of chemokines and their receptors in LPS-stimulated LA4 cells, qRT-PCR was performed using the custom RT^2^ Profiler PCR array in the same manner as CP treatments. Over- and under-expressed genes in the LPS-stimulated group compared with the normal group are listed in Table 3. Over- and under-expressed genes in the LPS-stimulated and OA 300 μM-treated group compared with the LPS-stimulated group are listed in Table 6. The levels of mRNA gene expression of CCL4, CXCL10, CXCL5, CCL5, CXCL3, CCL8, CCRL2, and PPBP (CXCL7) in the 300 μM OA-treated group were markedly down-regulated compared with those in the LPS-stimulated group. Over- and under-expressed genes in the LPS-stimulated and OA 100 μM-treated group compared with the LPS-stimulated group are listed in Table 7. The levels of mRNA gene expression of CXCL5, CCL4, CXCL3, CXCL7, CCL8, and CXCL1 in the 100 μM OA-treated group were significantly down-regulated compared with those in the LPS-stimulated group. In both groups of OA, 300 μM- or 100 μM-treated, the levels of mRNA gene expression of CCL4, CXCL5, CXCL3, CCL8, and CXCL7 were down-regulated in common compared with the LPS-stimulated group. The chemokines reversed by OA treatments—CCL4, CXCL3, and CXCL7—were found to be in line with the effects of CP treatments.

CXCL5, known as LIX (LPS-induced CXC chemokine in mice), can activate neutrophils and attract them into tissues during inflammation and infection via its receptor, CXCR2. CXCL5 was found to be associated with Th17 lymphocyte function. Liu et al. reported the enhanced expression of CXCL5 in differentiated alveolar epithelial type Ⅱ cells derived from human fetal lung and primary rodent type Ⅱ AEC culture in response to IL-17A/TNF-α and LPS stimulation, respectively [53]. CCL8, known as monocyte chemoattractant protein 2 (MCP-2), attracts and activates many immune cells, including basophils, eosinophils, mast cells, macrophages, and T cells, into inflammatory tissues and elicits their effects by binding to CCR1, CCR2, CCR3, and CCR5 [54,55]. Petrosino et al. showed the induced expression and release of CCL8 in both polyinosinic:polycytidylic acid (poly I:C)-treated HaCaT cells and ear keratinocytes from 2,4-dinitrofluorobenzene (DNFB)-induced murine contact allergic dermatitis. Poly I:C is an immunostimulant commonly used in immune system research [56].

In addition to the genes commonly down-regulated by OA treatments, the levels of mRNA gene expression of CXCL10, CCL5, and CCRL2 were down-regulated in the 300 μM OA-treated group, and the gene expression level of CXCL1 was down-regulated in the 100 μM OA-treated group. Concerning CCRL2, a detailed explanation of its decreased expression is covered in the Discussion section.

CCL5, known as RNATES (regulated on activation, normal T cells expressed and secreted), is chemotactic for monocytes, eosinophils, basophils, and T lymphocytes. CCL5 was identified as having a relationship with Th2 cells in the pathogenesis of respiratory allergic diseases, particularly regarding its association with inflammation in asthma [55,57].

In the CXC subfamily, the orthologs of CXCL5, CXCL4, CXCL3, and CXCL1 in mice correspond to CXCL6, CXCL4L1, CXCL1, and CXCL3 in humans, respectively. Indeed, mouse CXCL5 is functionally closer to human CXCL6. CXCL15 exists in mice but not in humans, whereas human CXCL8 does not have a mouse counterpart. Analyses indicate that CCL13 and CCL14 exist in humans but not in mice, while the mouse homologs of human CCL15 and CCL23 are CCL9 and CCL6, respectively [42,44]. Mouse CXCL1, CXCL2, and CXCL5 are regarded as functional homologs of human CXCL8. They have been found to contribute to the pathology of some neutrophil-dependent animal models of disease [58].

CXCL1 was found to be associated with Th17 lymphocytes. Its cytokine, IL-17, promotes the expression of CXCL1 and CXCL5 in bronchial epithelial cells, which leads to neutrophil production and recruitment [59]. Upregulated IL-17, CXCL1, and CXCL2 and increased neutrophil recruitment in a murine model of DNFB-induced contact hypersensitivity treated with lysophosphatidylcholine contributed to allergic skin inflammation [60]. CXCL1 expression was raised in the BAL of cockroach-sensitized mice and primary dendritic cells activated by house dust mites [61,62].

Our results showed that OA treatments displayed specific inhibitory effects on chemokines CCL4, CXCL10, CXCL5, CCL5, CXCL3, CCL8, CCRL2, CXCL7, and CXCL1. OA treatments could exhibit anti-inflammatory effects by controlling the recruitment of neutrophils, basophils, eosinophils, mast cells, macrophages, T cells, monocytes, and dendritic cells and by modulating Th1/Th2/Th17 lymphocyte functions based on the roles of the chemokines reversed by OA treatments.

### 3.2. Anti-Inflammatory Effects and Mechanisms of CP and OA Treatments in Lung Epithelial Cells

#### 3.2.1. Cell Viability Assay

**Figure 1:**
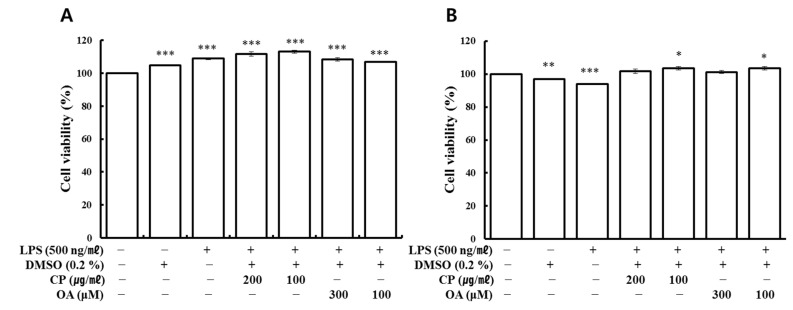
Cell viability by WST assay. (**A**) LA4 and (**B**) MH-S cells were treated at the indicated concentrations of the test compounds for 24 h. Untreated cells (normal) were set to 100%. Cell viabilities are expressed as a percentage relative to the normal group. The data represent the mean ± SEM of triplicate determinations. * *p* < 0.05, ** *p* < 0.01, and *** *p* < 0.001 vs. the normal group.

The cytotoxicity test was first performed for the subsequent experiments on the anti-inflammatory effects and mechanisms of CP and OA treatments. Cell viability was assessed by the water-soluble tetrazolium salt (WST) assay. There were no critical test compound-dependent effects in LA-4 and MH-S cells on cell viability, as shown in Figure 1A,B.

#### 3.2.2. Modulatory Effects of CP and OA Treatments on mRNA Gene Expression of Inflammatory Chemokines and Cytokines in LPS-Stimulated LA-4 Cells Using qRT-PCR

**Figure 2:**
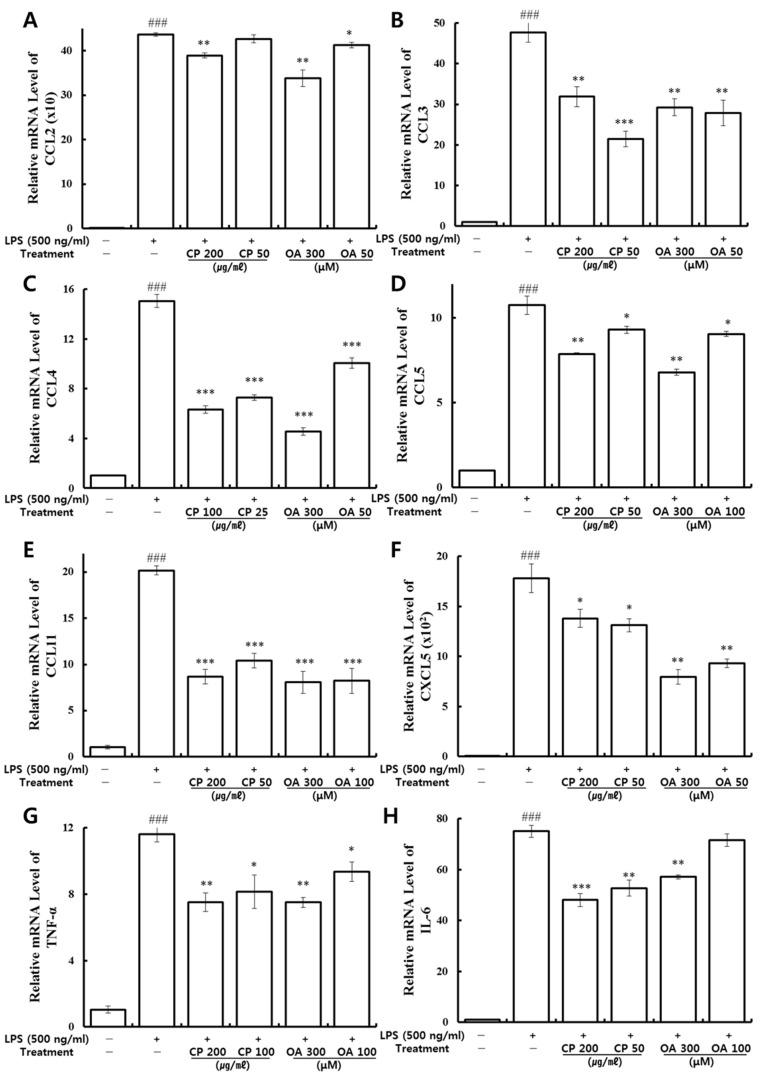
Effects of CP and OA treatments on mRNA gene expression of CCL2, CCL3, CCL4, CCL5, CCL11, CXCL5, TNF-α, and IL-6 in LPS-stimulated LA4 cells. (**A**) CCL2, (**B**) CCL3, (**C**) CCL4, (**D**) CCL5, (**E**) CCL11, (**F**) CXCL5, (**G**) TNF-α, and (**H**) IL-6 mRNAs were analyzed by qRT-PCR. The data are expressed as relative quantifications compared to the untreated group. The results are presented as the mean ± SEM of triplicate experiments. ### *p* < 0.001 vs. the untreated group; * *p* < 0.05, ** *p* < 0.01 and *** *p* < 0.001 vs. the LPS-stimulated group.

To further investigate the effects of CP and OA treatments on the mRNA gene expression of inflammatory chemokines and cytokines in LPS-stimulated LA4 cells, qRT-PCR was conducted with the synthesized mouse primers (Table 1). The mRNA gene expression levels of CCL2, CCL3, CCL4, CCL5, CCL11, CXCL5, TNF-α, and IL-6 were analyzed. As shown in Figure 2, they were significantly over-expressed by LPS stimulation. Then, those high expressions were significantly suppressed by CP treatments, with the exception of CCL2 expression, which was slightly decreased in the low-dose treatment (50 μg/mL). Likewise, OA (300, 100, and 50 μM) treatments markedly downregulated the mRNA expressions of CCL2, CCL3, CCL4, CCL5, CCL11, CXCL5, TNF-α, and IL-6, with the exception of IL-6 expression, which was slightly decreased in the 100 μM treatment group. CP and OA treatments significantly inhibited the mRNA gene expressions of CCL2, CCL3, CCL4, CCL5, CCL11, CXCL5, TNF-α, and IL-6, mainly at high concentrations, but also at a lower dose.

PRR activation leads to the induction, production, and release of pro-inflammatory mediators, such as the MAPK/NF-κB pathway. The pro-inflammatory mediators IL-6, TNF-α, and CXCL8 consequently entail the infiltration of activated immune cells, such as mast cells, DCs, and lymphocytes, which are involved in the pathogenesis of asthma. The presence of toxins or pathogens leads to the production and rapid and early secretion of IL-6, TNF-α, CXCL8, CCL11, and CCL20 in airway epithelial cells [17,63]. TNF-α is present in the infected environment within a few hours and then induces the expression of chemokines in epithelial cells [56]. It has been demonstrated that TNF-α stimulates the expression of CCL2, CCL4, CCL5, CCL11, and CCL20 [18]. Increased production of pro-inflammatory cytokines such as TNF-α, IL-4, and IL-13 has a disruptive effect on airway epithelial barrier function [17,64,65]. Additionally, the cytokines IL-4, IL-5, IL-13, CCL2, TNF-α, and IL-6 were significantly increased in the BALF of the OVA-induced airway inflammation model [66].

Rhinoviruses (RV) are a common trigger of exacerbations in asthma patients. Epithelial cells infected with RV produced inflammatory mediators, such as IL-6, CCL3, CCL5, CCL11, CXCL1, CXCL5, and CXCL8 [67]. These chemokines lead to the recruitment of macrophages, T cells, NK cells, eosinophils, and neutrophils, which facilitate the clearance of the virus. However, they may also amplify pre-existing airway inflammation [55]. In particular, neutrophils are recruited via CXCR2 by CXCL5 [17,18].

Some cell types, such as macrophages, dendritic cells, and B cells, express IL-6. This cytokine is involved in the acute phase response, macrophage differentiation, and B cell maturation. In asthma, the novel function of IL-6 is the control of Th1/Th2 differentiation. With the presence of IL-6, the Th1/Th2 balance is shifted towards the Th2 direction using two independent approaches. IL-6 activates the nuclear factor of activated T cells (NFAT), which induces the endogenous production of IL-4, leading to Th2 differentiation. On the other hand, IL-6 inhibits Th1 differentiation by interfering with IFN-γ signaling through the upregulation of SOCS1 [68].

The airway epithelium expresses CCL2, CCL3, CCL4, CCL5, CCL11, and CXCL5, which are associated with asthma. CCL2, known as monocyte chemotactic protein 1 (MCP-1), preferentially induces the accumulation and activation of monocytes in the inflamed area [38]. CCL2 also attracts monocytes, T lymphocytes, basophils, and NK cells via its receptors, CCR2 and CCR10 [17,18]. CCL2 is expressed by various cell types, including epithelial cells and macrophages [55]. A previous study investigated whether the blocking of CCL2 or neutralizing of CCR2 reduced the proportion of inflammatory monocytes and eosinophils in the BALF of an OVA-induced allergic mouse model [69]. CCL2 expression was upregulated in the OVA-induced acute asthmatic murine model, accompanied by significantly aggravated macrophage infiltration. Increased CCL2 expression was also observed in the LPS-stimulated human bronchial epithelial cells, followed by markedly upregulated macrophage migration [70]. CCL4 attracts lymphocytes and monocytes through CCR5 and CCR8 receptors. CCL3 has specific chemotactic activities for macrophages, eosinophils, T lymphocytes, DCs, neutrophils, and NK cells via its receptors CCR1, CCR3, and CCR5. CCL5 acts on CCR1, CCR3, and CCR5 and recruits eosinophils, monocytes, memory T cells, CD4+T cells, and basophils. CCL11 is an attractant for eosinophils, Th2 cells, basophils, and mast cells via its receptors, CCR3 and CCR5 [17,18]. CCL11 induces a respiratory burst in eosinophils through CCR3 [41].

Airway epithelial cells express CCR3, which reacts to its ligands, such as CCL5 and CCL11, in asthmatics. CCL5 and CCL11 are particularly crucial attractants for eosinophils [55]. CCR3 is one of the most relevant chemokine receptors in asthma, and its ligands are CCL3, CCL5, CCL11, and CCL13 [41]. The levels of CCL2, CCL3, and CCL5 in the BALF and CCL11 in plasma were significantly higher in asthma patients than in control subjects [18,41,65]. Additionally, CCL3, CCL5, and CCL11 were strongly upregulated in the epithelial cells of asthmatics compared to those of healthy controls [55,64].

Our results demonstrated that CP and OA treatments have specific inhibitory effects on the inflammatory chemokines and cytokines CCL2, CCL3, CCL4, CCL5, CCL11, CXCL5, TNF-α, and IL-6, and thus have a relevant effect on anti-inflammation in LPS-stimulated LA4 cells. CP and OA treatments could exhibit anti-inflammatory effects by suppressing the recruitment of mast cells, dendritic cells, lymphocytes, monocytes, macrophages, NK cells, eosinophils, neutrophils, memory T cells, CD4+T cells, and basophils and by modulating Th1/Th2/Th17 lymphocytes functions based on the roles of the chemokines reversed by CP and OA treatments.

#### 3.2.3. Suppressive Effects of CP and OA Treatments on the Production of Inflammatory Chemokines in LPS-Stimulated LA4 Cells

Following the analysis of mRNA gene expression on inflammatory chemokines, the effects of CP and OA treatments on the production of CCL2, CCL5, CXCL1, CXCL5, and CXCL7 in the LPS-stimulated LA4 cells were further investigated by utilizing ELISA.

**Figure 3:**
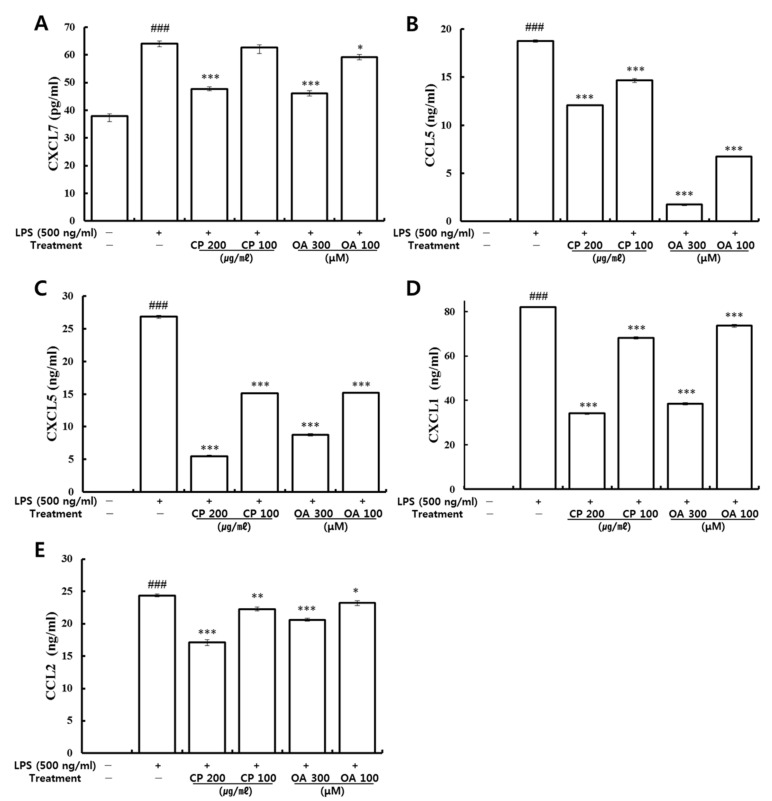
Effects of CP and OA treatments on the production of CXCL7, CCL5, CXCL5, CXCL1, and CCL2 in LPS-stimulated LA4 cells. Cells were stimulated with LPS for 1 h and followed by CP or OA treatments for 24 h. The secreted protein levels of (**A**) CXCL7, (**B**) CCL5, (**C**) CXCL5, (**D**) CXCL1, and (**E**) CCL2 were determined from the supernatant using an ELISA kit. The data are expressed as the mean ± SEM of triplicate experiments. ### *p* < 0.001 vs. the normal group; * *p* < 0.05, ** *p* < 0.01, and *** *p* < 0.001 vs. the LPS-stimulated group.

Previously, high expression levels, including those of CCL2, CCL3, CXCLl, CXCL2, CXCL10, and IL-6, were found in primary alveolar epithelial cells by LPS stimulation [26]. It was reported that a significant increase in inflammatory cytokines (IL-6 and TNF-α) and chemokines (CXCL1 and CCL2) occurred at the protein level with neutrophil accumulation in the BALF in the presence of LPS-induced lung injury in mice [71]. As shown in Figure 3, significantly higher levels of CCL2, CCL5, CXCL1, CXCL5, and CXCL7 proteins were produced in the LPS-stimulated group than in the normal group, which is consistent with the mRNA gene expression results (Table 3 and Figure 2). Then, CP treatment significantly suppressed the production of CCL2, CCL5, CXCL1, and CXCL5. Additionally, CP treatment (200 μg/mL) significantly inhibited the production of CXCL7, but there was only a slight decrease in the 100 μg/mL CP-treated group (Figure 3A). Levels of CCL2, CCL5, CXCL5, and CXCL7 were consistent with the mRNA gene expression results (Table 4 and Figure 2). Likewise, OA treatment significantly suppressed the secretion of CCL2, CCL5, CXCL1, CXCL5, and CXCL7, which is consistent with the results of mRNA gene expression (Table 6, Table 7, and Figure 2).

The fold regulations of CCL2 were excluded in Table 4, Table 5, Table 6 and Table 7 by the fold-regulation threshold cut-off value, which was set to 1.5. The fold regulations of CCL2 were marked as −1.20, −1.18, −1.08, and −1.14 in the complete lists in the tables (data not shown in the tables). For the same reason, the CCL5 fold regulations were expressed as −1.37, −1.36, and −1.38 in the complete lists in Table 4, Table 5 and Table 7, respectively. The fold regulations of CXCL1 were stated as −1.24, −1.14, and −1.09 in the complete lists in Table 4, Table 5 and Table 6, respectively. The fold regulations of CXCL5 were indicated as −1.42 and −1.36 in the complete lists in Table 4 and Table 5, respectively. Similarly, the CXCL7 (PPBP) fold regulation was shown as −1.41 in the complete list in Table 5.

Although the excluded chemokines were reported in those full lists in the tables with no significant differences, they all displayed decreasing patterns in their expression. Our results confirmed that CP and OA treatments have specific suppression effects on the protein production of CCL2, CCL5, CXCL1, CXCL5, and CXCL7.

#### 3.2.4. Inhibitory Effects of CP and OA Treatments on the Expression of PI3K/Akt, MAPKs, and NF-κB Pathways in LPS-Stimulated LA4 Cells

CP and OA treatments suppressed extensive pro-inflammatory chemokines and cytokines at mRNA and protein levels in the LPS-stimulated LA4 cells. Thus, we evaluated the impacts of CP and OA treatments on the inflammation-associated signaling pathways of nuclear factor-κB (NF-κB) p65, phosphatidylinositol 3-kinase (PI3K), Akt, and mitogen-activated protein kinases (MAPKs) in LPS-stimulated LA4 cells using Western blot.

PI3K is ubiquitously expressed in airway epithelial cells. The PI3K pathway plays crucial roles in expressing and activating inflammatory mediators, inflammatory cell recruitment, immune cell function, airway remodeling, and corticosteroid insensitivity in chronic inflammatory respiratory disease [72,73]. Inhibition of the PI3K pathway was found to relieve airway inflammation and hyperresponsiveness in a murine model of OVA-induced allergic asthma [74,75]. Akt is a serine/threonine protein kinase, which can be phosphorylated and activated by extracellular factors in the PI3K/Akt pathway [76]. In LPS-stimulated human lung epithelial cells, significantly increased expressions of PI3K/Akt, MAPKs, and NF-κB were observed [77,78], which are consistent with our results.

**Figure 4:**
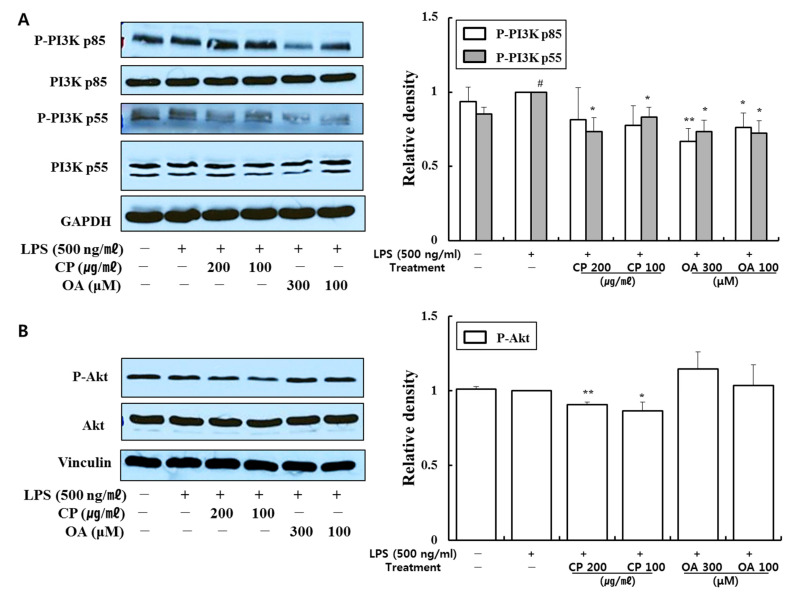
Effects of CP and OA treatments on the cellular protein levels of the PI3K/Akt pathway in LPS-stimulated LA4 cells. LA4 cells were stimulated with LPS for 1 h and then incubated with CP or OA treatments for 24 h. The proteins of (**A**) P-PI3K p85 (Tyr458), P-PI3K p55 (Tyr199), total PI3K p85, total PI3K p55, and (**B**) P-Akt (Ser473), total Akt were measured by Western blot. The images shown are representative, and GAPDH and vinculin were used as internal controls. The densities of the bands were assessed by Image J. The data are presented as the mean ± SEM; # *p* < 0.05 vs. the normal group; * *p* < 0.05 and ** *p* < 0.01 vs. the LPS-stimulated group. P-, phosphorylated.

**Figure 5:**
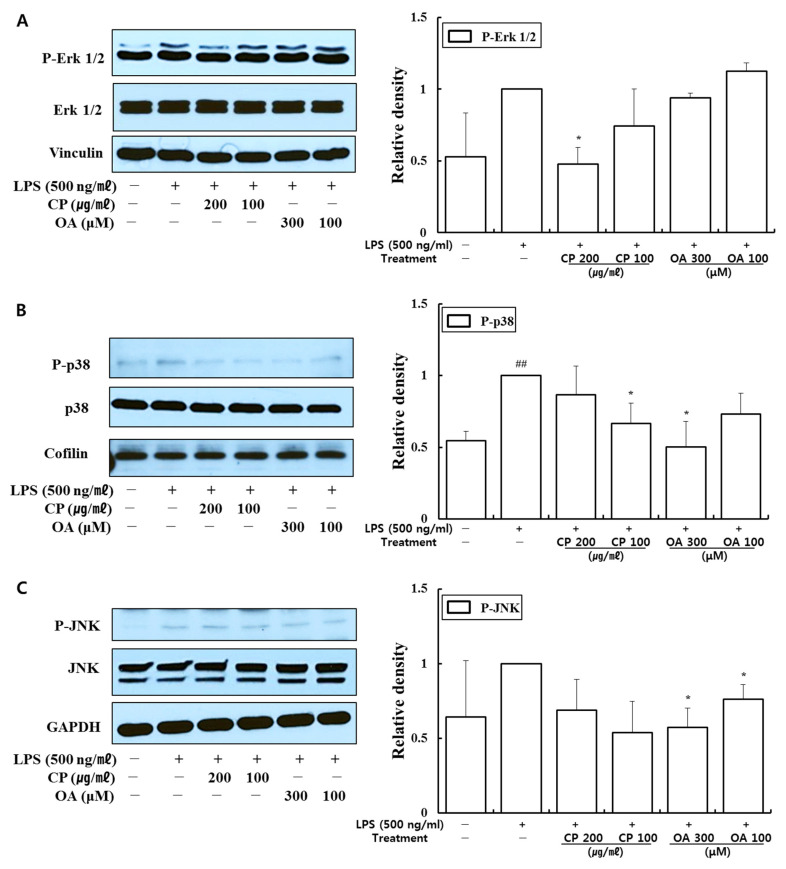
Effects of CP and OA treatments on the cellular protein levels of MAPKs in LPS-stimulated LA4 cells. LA4 cells were stimulated with LPS for 1 h and then incubated with CP or OA treatments for 24 h. The proteins of (**A**) P-Erk1/2 (Thr202/Tyr204), total Erk1/2, (**B**) P-p38 (Thr180/Tyr182), total p38, and (**C**) P-JNK (Thr183/Tyr185), total JNK were detected by Western blot. Vinculin, cofilin, and GAPDH were used as internal controls. The images shown are representative, and the densities of the bands were assessed by Image J. The data are indicated as the mean ± SEM; ## *p* < 0.01 vs. the normal group; * *p* < 0.05 vs. the LPS-stimulated group. P-, phosphorylated.

**Figure 6:**
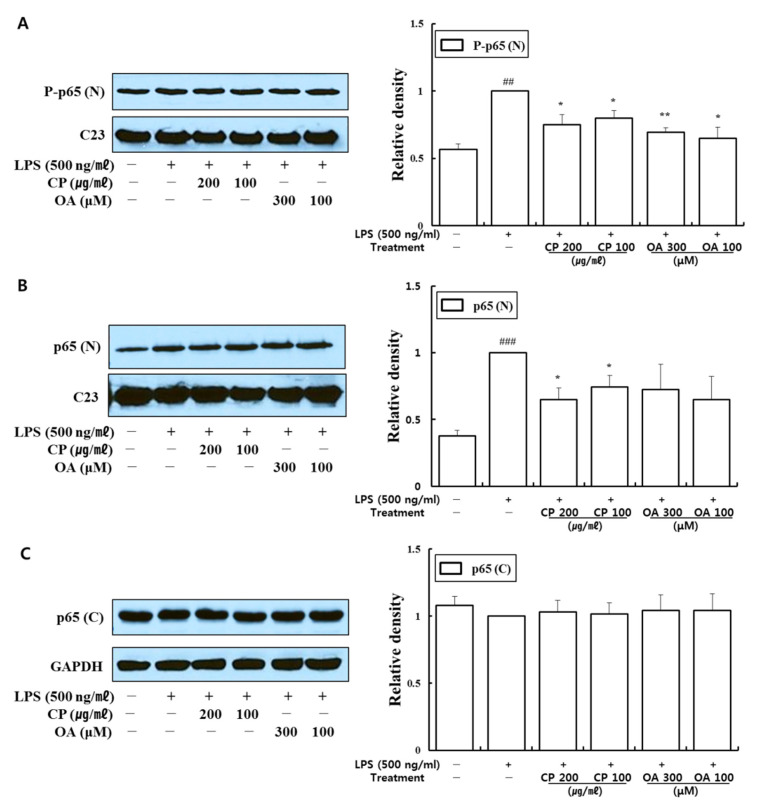
Effects of CP and OA treatments on the cellular protein level of NF-kB p65 in LPS-stimulated LA4 cells. LA4 cells were stimulated with LPS for 1 h and then incubated with CP or OA treatments for 24 h. Each protein fractioned into nuclear (N) and cytosol (C) was determined by Western blot. C23 was used for (**A**) P-p65 (Ser536) and (**B**) p65, and GAPDH was used for (**C**) p65 as internal controls. The images shown are representative, and the densities of the bands were assessed by Image J. The data are expressed as the mean ± SEM; ## *p* < 0.01, ### *p* < 0.001 vs. the normal group; * *p* < 0.05 and ** *p* < 0.01 vs. the LPS-stimulated group. P-, phosphorylated.

Our data demonstrated that CP treatments decreased the phosphorylation of PI3K-p85, PI3K-p55, Akt, extracellular signal-regulated kinase (Erk)1/2, p38, and c-Jun N-terminal kinase (JNK) (Figure 4 and Figure 5). In particular, CP treatments significantly suppressed the phosphorylations of PI3K-p55 (in 200 and 100 μg/mL-treated groups), Akt (in 200 and 100 μg/mL-treated groups), Erk1/2 (in 200 μg/mL-treated group), and p38 (in 100 μg/mL-treated group) compared with those in the LPS-stimulated group (Figure 4A,B and Figure 5A,B). CP treatments also significantly suppressed the phosphorylation and nuclear translocation of NF-κB p65 in the 200 μg/mL- and 100 μg/mL-treated groups compared to the LPS-stimulated group (Figure 6A,B). Likewise, OA treatments significantly suppressed the phosphorylations of PI3K-p85, PI3K-p55, p38, and JNK (Figure 4A and Figure 5B,C), with the exception of P-p38 expression, which was slightly decreased in the low-dose treatment (100 μM). OA treatments (300 and 100 μM) also significantly suppressed the phosphorylation of NF-κB-p65 and its nuclear translocation compared with those in the LPS-stimulated group (Figure 6A).

These results provide evidence that CP treatments exhibited anti-inflammatory effects on the inflammatory chemokines and cytokines by inhibiting pro-inflammatory mediators, including PI3K-p55, Akt, Erk1/2, p38, and NF-κB, in LPS-stimulated LA4 cells. Likewise, OA treatments displayed anti-inflammatory effects on the inflammatory chemokines and cytokines by inhibiting pro-inflammatory mediators, including PI3K-p85, PI3K-p55, p38, JNK, and NF-κB, in LPS-stimulated LA4 cells.

### 3.3. Anti-Inflammatory Effects and Mechanisms of CP and OA Treatments in Lung Macrophages

#### 3.3.1. Modulatory Effects of CP and OA Treatments on mRNA Gene Expression of Inflammatory Chemokines and Cytokines in LPS-Stimulated MH-S Cells Using qRT-PCR

**Figure 7:**
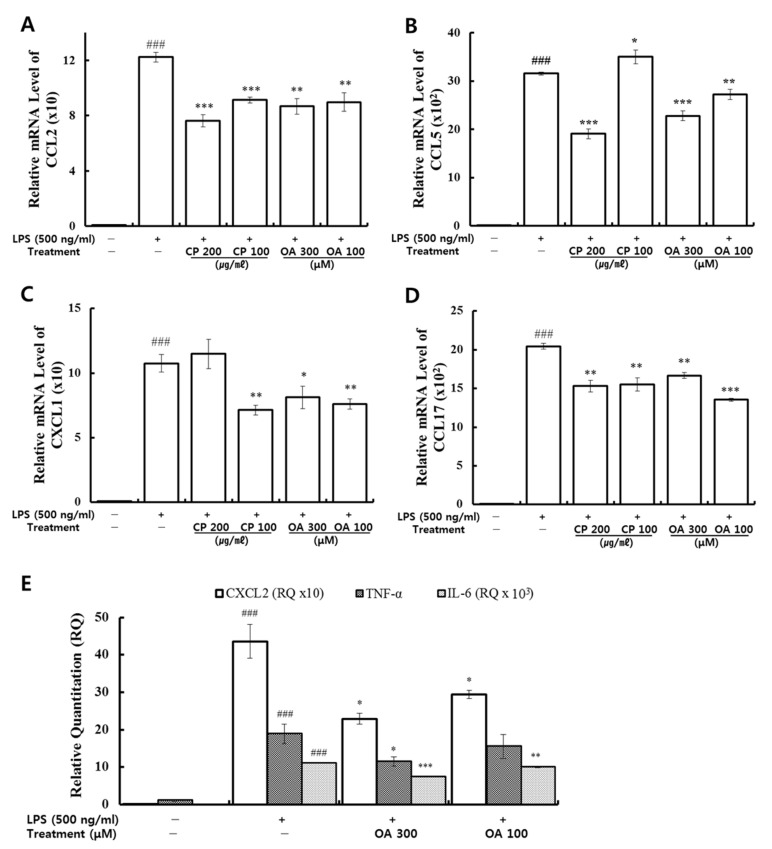
Effects of CP and OA treatments on mRNA gene expression analyzed by qRT-PCR in LPS-stimulated MH-S cells. The mRNA levels of (**A**) CCL2, (**B**) CCL5, (**C**) CXCL1, (**D**) CCL17, (**E**) CXCL2, TNF-α, and IL-6 are expressed as the relative quantitation (RQ) to the untreated group. The results are presented as the mean ± SEM of triplicate experiments. ### *p* < 0.001 vs. the untreated group; * *p* < 0.05, ** *p* < 0.01 and *** *p* < 0.001 vs. the LPS-stimulated group.

Lung macrophages act as the first line of phagocytic defense against pathogen invasion into the lower respiratory tract. Noteworthily, macrophages are responsible for regulating inflammatory responses in the lungs by secreting numerous chemical mediators upon stimulation [79]. Therefore, we further investigated the effects of CP and OA in lung macrophages. qRT-PCR was conducted using mouse primers (Table 1) to measure the mRNA gene expression of inflammatory chemokines and cytokines in LPS-stimulated MH-S cells. The expressions of CCL2, CCL5, CXCL1, CXCL2, CCL17, TNF-α, and IL-6 were analyzed. As shown in Figure 7, they were markedly over-expressed after LPS stimulation. Then, the high expression levels of CCL2 and CCL17 were significantly suppressed by CP treatment. The expression levels of CCL5 (200 μg/mL CP treatment) and CXCL1 (100 μg/mL CP treatment) were markedly reduced compared to the LPS-stimulated group. Similarly, OA treatments significantly inhibited the expression levels of CCL2, CCL5, CCL17, CXCL1, CXCL2, and IL-6 compared to those in the LPS-stimulated group. The 300 μM OA treatment also markedly suppressed the expression of TNF-α. We can therefore confirm the additional inhibitory effects of OA treatment on the expressions of CXCL2, TNF-α, and IL-6.

LPS, one of the potent stimulants directionally inducing M1 polarization, activates pro-inflammatory cytokines and chemokines such as CCL2, CCL5, CXCL1, CXCL2, IL-6, and TNF-α [32,33], which are relevant to our experiment. Tissue macrophages directly synthesize the major neutrophil chemoattractants CXCL1 and CXCL2, which bind to CXCR2 in response to LPS, mainly through TLR4 in a dependent manner. Recruitment of neutrophils is an essential early step to control tissue infection or damage in the onset of the innate immune response to microbial challenge. Those neutrophil responses to macrophages are CXCL1/2-dependent, as demonstrated by in vitro chemotaxis experiments and in vivo studies [31,80].

The neutralization of CCR2 (the receptor for CCL2) on macrophages attenuated macrophage accumulation in the BALF and reduced antigen-induced bronchial hyperresponsiveness in asthmatic monkeys [81], while airway allergen challenge in patients with mild allergic asthma led to high levels of CCR2 expression [31]. Previous studies demonstrated that blocking the CCL2/CCR2 axis markedly reduced Th17 levels and revealed an inhibitory effect on airway inflammation [82].

IL-4- or IL-13-induced M2a macrophages typically express CCL17 [83]. However, it was shown that CCR4 (the receptor for CCL17)-dependent CCL17 plays a role in M1 activation of lung-associated and monocyte-derived macrophages, leading to iNOS induction. Meanwhile, CCR4-deficient (CCR4−/−) macrophages more frequently displayed the M2 phenotype [84]. In addition, CCR4−/− mice showed lower serum levels of TNF-α and CCL3 after LPS administration than CCR4 WT mice, which suggests that CCR4 plays a specific role in the process of LPS invasion [85]. Macrophages in asthmatics’ sputum exhibited significantly upregulated CCL17 mRNA expression, which highly correlated with sputum eosinophilia, but other M2 biomarkers were not differentially expressed in asthmatic individuals [86]. Additionally, CCR4 controls the homeostatic function of M1/M2 macrophage differentiation and promotes macrophage phagocytosis and respiratory burst [41].

CCL5 mediates neutrophil, monocyte, and eosinophil chemotaxis to the airway, and its production is associated with airway inflammation [87,88]. The affinity of CCL5 binding for its ligands, CCR1 and CCR5, is significantly greater than CCR3. Th2 cells express CCR3, and Th1 cells express CCR1 and CCR5 [16,88]. Mycobacterial-induced Th1-type lesions showed higher CCL5 mRNA and protein expressions than schistosomal-induced Th2-type lesions in a pulmonary granuloma mouse model [89]. However, CCL5 modulates cytokine production, such as inducing the upregulation of IFN-γ (Th1 cytokine), IL-5 and IL-6 (Th2 cytokines), so whether CCL5 contributes to Th1 cell- or Th2 cell-mediated responses depends on the situation [90]. In mice, CCL5 was upregulated in parallel with a decline in airway hyperresponsiveness after repeated allergen challenge [87]. Meanwhile, CCL5 is constitutively expressed in asthmatics’ lungs, and its chemotaxis for eosinophils through the CCR3 receptor has been identified. Much evidence suggests that CCL5 is expressed in asthmatic patients, and its expression correlates with disease severity. Focusing on CCL5 and its receptors could be potential therapeutic target in asthmatics [88], even though the working mechanism of CCL5 in the Th1/Th2 balance appears to be complex.

M1 macrophages release high levels of pro-inflammatory cytokines, such as TNF-α and IL-6, to deal with infections, which is consistent with the LPS stimulation results in our data. We confirmed that OA treatments significantly reduced the expression levels of IL-6 and TNF-α. IL-6 is associated with the development and expansion of Th17 cells. IL-6 and TGF-β can develop Th17 cells in mice, whereas in humans, they might be developed in the presence of IL-1β and IL-6 [91]. The role of TNF-α between macrophages and alveolar epithelial cells using co-culture methods has been demonstrated. TNF-α production by hypoxia–reoxygenation of exposed RAW264.7 cells significantly induced CXCL1 and CXCL2 expression in lung epithelial cells (MLE-12), which may significantly contribute to lung injury by recruiting neutrophils [92,93].

Our data demonstrated that CP treatments exhibited specific inhibitory effects on the mRNA gene expression of inflammatory chemokines CCL2, CCL5, CCL17, and CXCL1. CP treatments could display anti-inflammatory effects by suppressing the recruitment of macrophages, neutrophils, monocytes, and eosinophils and by modulating Th1/Th2/Th17 lymphocytes’ functions based on the roles of the chemokines reversed by CP treatments. OA treatments exhibited more extensive inhibitory effects on the mRNA gene expression of the inflammatory chemokines and cytokines CCL2, CCL5, CXCL1, CCL17, CXCL2, TNF-α, and IL-6. OA treatments could display anti-inflammatory effects by suppressing the recruitment of macrophages, eosinophils, neutrophils, and monocytes and by modulating Th1/Th2/Th17 lymphocytes based on the roles of the chemokines reversed by OA treatments.

#### 3.3.2. Suppressive Effects of CP and OA Treatments on the Production of Inflammatory Chemokines in LPS-Stimulated MH-S Cells

**Figure 8:**
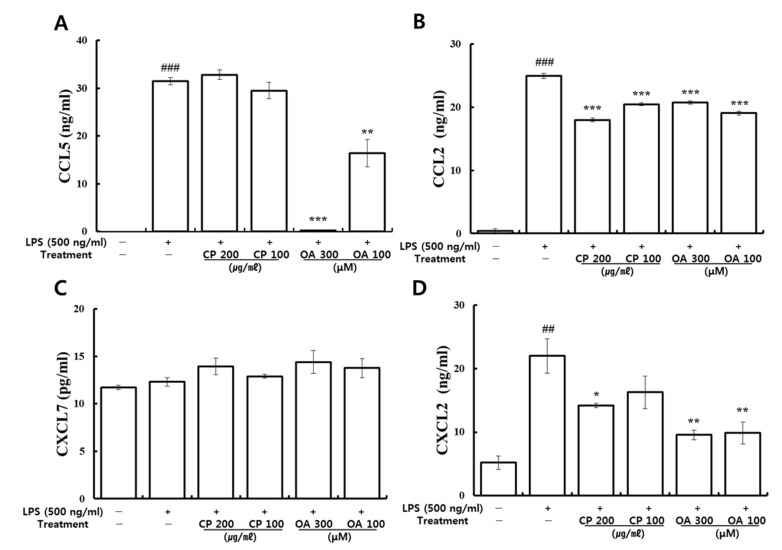
Effects of CP and OA treatments on the production of CCL5, CCL2, CXCL7, and CXCL2 in LPS-stimulated MH-S cells. Cells were stimulated with LPS for 1 h and followed by treatments of CP or OA for 24 h. The secreted protein levels of (**A**) CCL5, (**B**) CCL2, (**C**) CXCL7, and (**D**) CXCL2 were determined from the supernatant using an ELISA kit. The data are expressed as the mean ± SEM of triplicate experiments. ## *p* < 0.01, ### *p* < 0.001 vs. the normal group; * *p* < 0.05, ** *p* < 0.01, and *** *p* < 0.001 vs. the LPS-stimulated group.

The effects of CP and OA treatments on the production of CCL2, CCL5, CXCL2, and CXCL7 in LPS-stimulated MH-S cells were further investigated by utilizing ELISA. As shown in Figure 8, significantly higher protein levels of CCL2, CCL5, and CXCL2 were produced in the LPS-stimulated group than in the normal group. Then, the expression level of CCL2 was significantly reduced by CP treatments. CXCL2 expression was markedly reduced by the high-dose CP treatment (200 μg/mL) compared to the LPS-stimulated group. However, there were no significant inhibitions in the production of CCL5 by CP treatments. Meanwhile, OA treatments significantly inhibited the production of CCL2, CCL5, and CXCL2, which is consistent with the gene expression results. There were no significant changes in the production of CXCL7 by CP or OA treatments or by LPS stimulation.

Our results confirmed that OA treatments have specific suppression effects on the protein production of CCL2, CCL5, and CXCL2, and that CP treatments have specific suppression effects on the protein production of CCL2 and CXCL2.

#### 3.3.3. Inhibitory Effects of CP and OA Treatments on the Expression of PI3K, iNOS, and Cox-2 in LPS-Stimulated MH-S Cells

CP and OA treatments suppressed pro-inflammatory chemokine and cytokine expressions at mRNA and protein levels in MH-S cells. Thus, we evaluated whether CP and OA treatments affect PI3K p55, inducible nitric oxide synthase (iNOS), and cyclooxygenase (Cox)-2 expressions in LPS-stimulated MH-S cells using Western blot.

Macrophages can be induced to express iNOS and Cox-2 following LPS stimulation and can produce nitric oxide (NO) and prostaglandin E2 (PGE2) [94,95,96]. Many studies have found that increased iNOS and Cox-2 activities are associated with chronic inflammation [95]. In particular, excessive release of fractional exhaled nitric oxide (FeNO) is observed in experimental asthma models and asthma patients, and FeNO has been found to correlate with the severity of airway inflammation [97]. Significantly increased Cox-2 expression was detected in the lungs of an OVA-induced allergic asthma model, along with increased production of PGE2 in the bronchoalveolar lavage fluid (BALF) [96]. It has been shown that activation of the PI3K/Akt pathway, in turn, induces MAPK activation and initiates NF-κB activation [98]. The nuclear translocation of free NF-κB induces the transcription of pro-inflammatory mediators, such as iNOS and Cox-2 [99].

**Figure 9:**
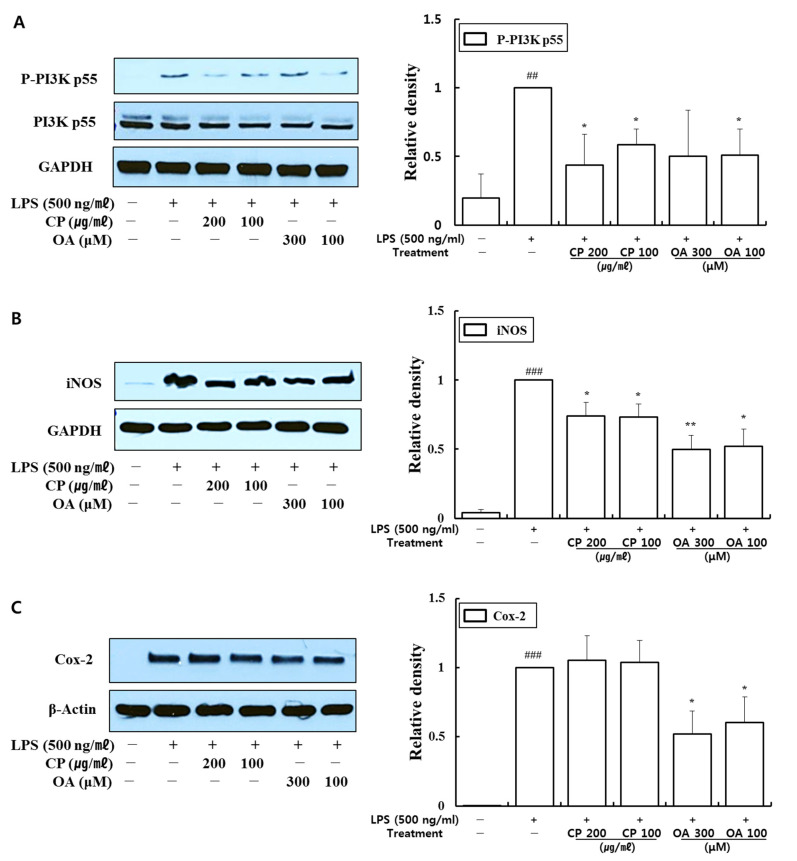
Effects of CP and OA treatments on the cellular protein levels of PI3K, iNOS, and Cox-2 in LPS-stimulated MH-S cells. MH-S cells were stimulated with LPS for 1 h and then incubated with CP or OA treatments for 24 h. The proteins of (**A**) phospho-PI3K p55 (Tyr199), total PI3K p55, (**B**) iNOS, and (**C**) Cox-2 were determined by Western blot. The images shown are representative, and GAPDH and β-actin were used as internal controls. The densities of the bands were assessed by Image J. The results are presented as the mean ± SEM; ## *p* < 0.01, ### *p* < 0.001 vs. the normal group; * *p* < 0.05 and ** *p* < 0.01 vs. the LPS-stimulated group. P-, phosphorylated.

As shown in Figure 9, LPS stimulation significantly provoked the expression of phospho-PI3K p55, iNOS, and Cox-2. CP treatments then significantly suppressed the expression of phospho-PI3K p55 and iNOS compared with those in the LPS-stimulated group. Likewise, OA treatments significantly suppressed the expression of iNOS and Cox-2, and OA 100 μM treatment also markedly inhibited phospho-PI3K p55 expression compared with the LPS-stimulated group. Notably, CP and OA treatments inhibited PI3K as a potential upstream regulator of iNOS and Cox-2 expressions.

These results provide evidence that CP treatments display anti-inflammatory effects on inflammatory chemokines by inhibiting pro-inflammatory mediators, including PI3K-p55 and iNOS, in LPS-stimulated MH-S cells. Likewise, OA treatments demonstrated anti-inflammatory effects on inflammatory chemokines and cytokines by suppressing pro-inflammatory mediators, including PI3K-p55, iNOS, and Cox-2, in LPS-stimulated MH-S cells.

## 4. Discussion

Chemokines convey information to leukocytes in the homeostatic or inflammatory environment and control the immune responses through chemokine receptors widely expressed on immune cells. Chemokines and chemokine receptors are intensively involved in innate and adaptive immune responses [41]. In this study, we aimed to assess the anti-inflammatory effects and mechanisms of CP and OA treatments in LPS-induced lung and airway inflammation in vitro. In particular, we conducted comprehensive gene profiling on the chemokines and their receptors in mouse lung epithelial cells. Then, we tried to characterize the classical Th2 and non-Th2 endotypes with chemokines discriminately from the classification using interleukins.

LPS is a highly immunogenic antigen with the ability to stimulate the host cells of the innate immune system via toll-like receptor (TLR) 4, which is one of the PRRs implicated in inflammatory pathways. TLR4 recognizes common PAMPs presented on phagocytic and epithelial cells [100,101]. This stimulation results in various pro-inflammatory cytokines and chemokines that mediate inflammation and is strongly related to lung damage [71,102]. Therefore, LPS stimulation of lung epithelial cells and lung macrophages in vitro can mimic lung inflammation. Lung epithelial cells are constantly exposed to microbial challenges due to respiration and are responsive to the TLR4 activator LPS [102,103]. LPS induced neutrophil accumulation and increased the expression of pro-inflammatory molecules in rodents [104], possibly by activating TLR4/NF-κB signaling pathways, leading to inflammation [104,105].

The response to LPS is dependent on binding to the membrane receptor CD14 in the presence of LPS binding protein (LBP) in serum. CD14, along with TLR4 and myeloid differentiation factor 2 (MD-2), functions as a co-receptor for LPS detection for signal transduction. TLR4, with CD14, plays a role in the cellular response to pathogens as the primary mediator of LPS signaling, followed by transcription factor NF-κB activation and cytokine production [106,107].

Interestingly, LPS stimulation significantly downregulated CXCL12 expression in our study (Table 3). CXCL12 acts via CXCR4 and ACKR3 receptors, which are significantly expressed on the cancer cell surface [108]. Classically, CXCL12 recruits stromal cells in the lungs under normal conditions, and CXCR4 has been implicated in more than 20 human cancers. As the CXCL12/CXCR4 axis correlates with the angiogenesis, proliferation, and metastasis of tumors, the axis is attracting increasing attention as a potential therapeutic target [109]. However, there is some evidence of the opposing functions of CXCL12, so it is somewhat controversial. Some suggested that a reason for this discrepancy may be due to the existence of at least six CXCL12 isoforms in humans and three isoforms in mice, each with a different role [45,108]. In our study, the decreased expression of CXCL12 in the LPS-stimulated group may be explained by the following two studies.

The response to LPS by the innate immune system is mainly dependent on TLR4, along with CD14 and MD-2. Additional receptors, such as CD55, CXCR4, and heat shock proteins (HSP), have been proposed as part of the activation cluster. Triantafilou et al. suggested that CXCR4 seems to be crucial for LPS signaling with other LPS co-receptors [110]. In addition, a previous study demonstrated that the expression levels of inflammatory factors such as TNF-α, IL-6, IL-8, COX-2, and NF-κB were downregulated by a CXCR4 antagonist drug [111]. Dai et al. emphasized the CXCL12/CXCR4 autocrine loop, which significantly promotes the motility, proliferation, and invasiveness of non-small cell lung cancer (NSCLC). The findings suggest that therapies related to CXCR4 in the treatment of NSCLC should consider CXCL12’s expression status. This is because autocrine overexpression of CXCL12 in some tumors may be more sensitive to CXCR4 antagonists by competing with CXCL12 for receptor binding, meaning that they may be more suitable for the application of chemokine-based anti-cancer therapies [112]. Therefore, in our study, LPS probably interfered with the autocrine expression of CXCL12 in the CXCL12/CXCR4 axis via competition for CXCR4 binding. Thus, LPS may downregulate CXCL12 expression, whereas CXCR4 may act as one of the co-receptors for LPS binding. Consequently, LPS may act as an antagonist to CXCR4. Scrutiny of those mechanisms would be an excellent topic for further study.

In the other study, the expression of CXCL12-γ in the central nervous system (CNS) was significantly decreased in experimental autoimmune encephalomyelitis (EAE)-prone Dark Agouti (DA) rats compared to that of EAE-resistant Albino Oxford (AO) rats. In CNS, inhibition of nitric oxide (NO) synthesis in DA rats upregulated CXCL12-γ expression, while the contribution of NO in AO rats downregulated CXCL12-γ expression. Moreover, NO remarkably inhibited CXCL12-γ expression in astrocytes in vitro, suggesting its modulatory effect on CXCL12-γ expression in neuroinflammation [113]. It can be assumed that a similar mechanism would have worked in our study as well since the expression of iNOS in LPS-stimulated macrophage cells was significantly increased compared with that in the normal group in the Western blot results (Figure 9). A similar pattern was also confirmed in our preliminary data for the lung epithelial cells (data not shown).

Asthma is also a multicellular disease because it involves integrative reactions to triggers in the airways, which cause abnormal or excessive responses by various cell types, including mast cells, eosinophils, dendritic cells, neutrophils, macrophages, T lymphocytes, and other airway constituent cells such as airway smooth muscle cells and airway epithelial cells [20,114]. The highest numbers of eosinophils and T lymphocytes were found in bronchial biopsies of allergic and nonallergic asthma [115]. CD4+ T cells can differentiate into one of several distinct T cell types functionally when they come across antigens. In particular, Th1 cells typically express CCR1, CCR5, CXCR3, and CXCR6; Th2 cells preferentially express CCR3, CCR4, and CCR8; and Th17 cells express CCR6. These effector T cells can migrate to inflamed tissues via their receptors, contributing to microbial clearance and tissue repair. In particular, CXCL9, CXCL10, and CXCL11 are associated with Th1 cell functions; CCL1, CCL17, CCL18, and CCL22 are associated with Th2 cell functions; and CXCL1, CXCL2, CXCL3, CXCL5, CXCL6, and CXCL8 are associated with Th17 cell functions. In addition, CCL4 was significantly associated with a mix of lymphocytes, including Th1, Th2, Th9, and Th17, in severe asthma patients [16,39]. Furthermore, expression levels of CCR3 are high in eosinophils. Eosinophil migration is mediated by a gradient of CCL3, CCL7, and CCL22, mainly produced by interstitial macrophages, and the gradient of these chemokines can be changed by the chemokines CCL5 and CCL11, which are released at high levels by the airway epithelium [55]. Despite the application of standard therapy with bronchodilators and inhaled corticosteroids, approximately 10% of asthmatics have persistent severe symptoms [20]. Given the vital roles of chemokines in the immune system and during inflammatory responses, a number of chemokines and GPCRs seem to have an essential contribution to the pathogenesis of asthma [116]. These molecules have become the most prominent targets for drug development because of their active participation in disease progression [109]. At present, many chemokine inhibitors are under development for the treatment of asthma. For example, anti-CCR3 (ASM8) and anti-CCL11 (Bertilimumab) are in phase 2 clinical trials [57].

Through LPS stimulation, LA4 cells (type Ⅱ-like AECs) express diverse chemokines, which can chemoattract various innate and adaptive immune cells [26,71,117,118]. The inhibition of inflammatory chemokines apparently affects the local inflammatory response by interfering with immune cell recruitment. In allergic asthma, a significant negative correlation was found between the numbers of T lymphocytes and eosinophils and epithelial integrity [115].

Targeted therapies that modulate cell signaling pathways can be a powerful strategy to treat asthma. Thus, signaling molecules can be potential targets for treating asthma. MAPK signaling pathways regulate immune responses and inflammation by controlling the gene expression of inflammatory factors in asthma. Fengjuan et al. reported significant increases in IgE and IL-4 and inflammatory mediators such as IL-6, IL-17, TNF-α, and NO in an OVA-induced rat asthma model. IL-4 and IgE could be regulated by inhibiting the phosphorylation of Erk, JNK, and p38, and the nuclear translocation of phospho-p65 [119,120]. The IL-4/IL-13/STAT-6 pathway is a well-known key regulator in asthma pathophysiology. MAPKs transactivate STAT-6 by phosphorylating its serine residues. In particular, the inhibition of STAT-6 phosphorylation by Erk and p38 inhibitors suppressed the expression of IL-4/IL-13-induced inflammatory chemokines such as CCL2, CCL11, CXCL1, and CXCL3. Thus, inhibitors of Erk and p38 can be considered as potential therapeutic agents in asthma [119]. JNK also plays a meaningful role in the airway remodeling and apoptotic process by inducing the Wnt5a/JNK signaling pathway in asthma [121]. Furthermore, considerable research efforts have revealed that the phosphorylation of NF-κB contributes to controlling NF-κB-directed transactivation. Crucially, NF-κB phosphorylation provides new opportunities to selectively target NF-κB as therapeutic targets by controlling transcription in a gene-specific manner [122]. Indeed, previous studies have found that the phosphorylation of p65 at S536 is not regulated by, or associated with, IκBα. Instead, this phosphorylation may lead to the expression of a distinct set of target genes [122,123]. In addition, the PI3K/Akt signaling pathway contains upstream molecules of NF-κB, which has a regulatory role in allergic asthma [97,124,125,126,127].

In our study, CP treatments probably exhibit their anti-inflammatory effects by suppressing the recruitment of mast cells, dendritic cells, NK cells, monocytes, macrophages, eosinophils, neutrophils, basophils, and lymphocytes, and by modulating T cell differentiation, dendritic cell maturation, and Th1/Th2/Th17 lymphocytes according to the roles of the reversed chemokines (Table 4 and Table 5, Figure 2 and Figure 3), as described in the results. These results prove that CP treatments exhibit anti-inflammatory effects on suppressed inflammatory chemokines and cytokines by inhibiting pro-inflammatory mediators, including PI3K-p55, Akt, Erk1/2, p38, and NF-κB, in the LPS-stimulated LA4 cells (Figure 4, Figure 5 and Figure 6).

Likewise, OA treatments possibly exhibit anti-inflammatory effects by suppressing the recruitment of mast cells, dendritic cells, NK cells, neutrophils, basophils, eosinophils, monocytes, macrophages, lymphocytes, and by modulating Th1/Th2/Th17 lymphocytes functions according to the roles of the reversed chemokines (Table 6 and Table 7, Figure 2 and Figure 3), as described in the results. These results prove that OA treatments display anti-inflammatory effects on inflammatory chemokines, cytokines, and a receptor by inhibiting pro-inflammatory mediators, including PI3K-p85, PI3K-p55, p38, JNK, and NF-κB, according to the data (Figure 4, Figure 5 and Figure 6). It is notable on the mechanisms of action of CP and OA concerning the inhibition of PI3K.

Interesting studies have been published regarding the inhibition of CCRL2 by OA treatment (Table 6). CCRL2 belongs to the group of ACKRs. ACKRs are devoid of chemotactic activity and are characterized by their ability to scavenge chemotactic factors from inflamed tissues. Despite being in the group of ACKRs, CCRL2 does not bind chemokines and is devoid of scavenging functions, but does modulate leukocyte migration. CCRL2 is expressed by monocytes/macrophages, mast cells, dendritic cells, bronchial epithelia, endothelial cells, microglia, and astrocytes in mice. In studies, CCL2, CCL5, CCL7, CCL8, and CCL19 were proposed as its ligands, but this was not subsequently confirmed. Thus far, only chemerin, a non-chemokine chemotactic protein, has been generally accepted as a CCRL2 ligand. CCRL2 remains unexplored in various aspects, but its roles in relation to inflammation, leukocyte migration, and tumors have been reported [128,129]. The expression of CCRL2, which has been reported to form a heterodimer with CXCR2, was rapidly upregulated by LPS, TNF, and other inflammatory stimuli in neutrophils and dendritic cells and induced in lung macrophages and the bronchial epithelia in OVA-induced airway inflammation [130,131]. Otero et al. demonstrated the unique role of CCRL2 in lung DC migration using CCRL2 KO mice. CCRL2 KO mice showed a marked reduction in leukocyte recruitment in the bronchoalveolar lavage, particularly with regard to mononuclear cells and eosinophils in a Th2 model of OVA-induced airway inflammation, whereas regular recruitment of circulating DCs into the lungs did occur. This reduction in leukocyte recruitment was caused by decreased production of Th2 cytokines (IL-4 and IL-5) and chemokines (CCL11 and CCL17). The decreased local Th2 response was directly correlated with the reduced migration of antigen-loaded lung DCs to mediastinal lymph nodes and the subsequently decreased priming of antigen-specific T cells in the regional lymph nodes. The study showed the specific role of CCRL2 in lung DCs in terms of their migration into mediastinal lymph nodes and their contribution to excessive lung inflammation [132].

According to traditional Korean medicine, CP exhibits its efficacy in the lungs and liver. Interestingly, OA, one of the main bioactive components in CP, has shown a specific inhibitory effect on CCRL2 expression in LPS-stimulated epithelial cells (Table 6). OA treatments may interrupt neutrophil recruitment by decreasing CCRL2. It is possible that OA exerts its inhibitory effect on CCRL2 expression in lung DCs and subsequently contributes to the resistance to allergic asthma by decreasing the migration of lung DCs into mediastinal lymph nodes. Additional follow-up studies investigating the OA mechanism concerning CCRL2 in animal models would be intriguing.

Unbalanced polarization or overall activation of macrophages contributes to chronic lung inflammation in asthma, as with Th1/Th2 polarization of T cells. The complex networks of chemokines and their receptors actively participate in macrophage polarization and activation [32,33,133]. Macrophages have been extensively acknowledged as desirable therapeutic targets for inflammatory diseases [33].

The expressions of iNOS and Cox-2 are upregulated by activated NF-κB, which are crucial mediators in the development of pulmonary inflammation. Meanwhile, the activation of NF-κB by iNOS and Cox-2 can subsequently induce other inflammatory cells and mediators. Therefore, the regulation of iNOS and Cox-2 is required to control inflammation in the airways and lungs [119].

In our study, CP treatments probably exhibit anti-inflammatory effects by suppressing the recruitment of macrophages, neutrophils, monocytes, and eosinophils and modulating Th1/Th2/Th17 lymphocytes according to the roles of the reversed chemokines (Figure 7 and Figure 8), as described in the results. These results prove that CP treatments exhibit anti-inflammatory effects on suppressed inflammatory chemokines (Figure 7 and Figure 8) by inhibiting pro-inflammatory mediators, including phospho-PI3K p55, iNOS, and Cox-2, in LPS-stimulated MH-S cells (Figure 9). Previous studies showed the suppressed expression of iNOS and Cox-2 by a PI3K inhibitor in the bronchial epithelia of asthmatic rats and particulate matter (PM)-exposed bronchial epithelia, respectively [97,134]. Our data may indicate that CP treatments exhibited suppressive effects on iNOS expression by downregulating the phosphorylation of PI3K p55. Likewise, OA treatments could exhibit anti-inflammatory effects by inhibiting the recruitment of macrophages, eosinophils, and neutrophils, monocytes, and by modulating Th1/Th2/Th17 lymphocytes according to the roles of the reversed chemokines and cytokines (Figure 7 and Figure 8), as described in the results. These results prove that OA treatments demonstrate anti-inflammatory effects on inflammatory chemokines and cytokines by suppressing pro-inflammatory mediators, including phospho-PI3K p55, iNOS, and Cox-2, in LPS-stimulated MH-S cells (Figure 9). Our data may indicate that OA treatments exhibited suppressive effects on iNOS and Cox-2 expression by downregulating the phosphorylation of PI3K p55 according to previous studies [97,134]. Notably, CP and OA treatments inhibited PI3K as a potential upstream regulator of iNOS and Cox-2 expression in LPS-stimulated MH-S cells.

**Figure 10:**
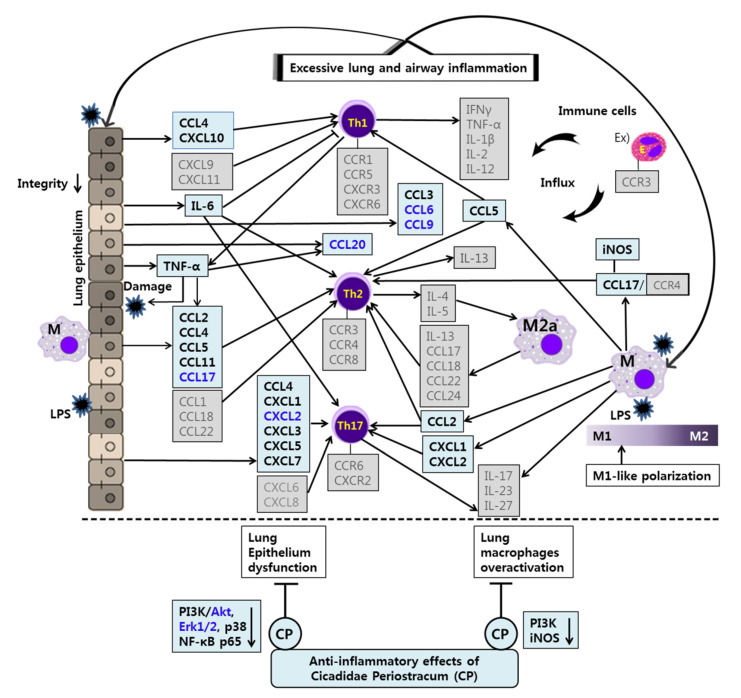
Schematic illustration of the anti-inflammatory effects and mechanisms of CP treatments in LPS-induced lung and airway inflammation in vitro. The lung epithelial cells and macrophages over-expressed extensive inflammatory chemokines and cytokines due to LPS stimulation, which leads to the recruitment of various immune cells and subsequently causes excessive lung and airway inflammation seen in asthmatics. The data in the gray boxes were cited from [16,17,18,20,21,32,33,39,50,51,52,53,55,58,59,63,64,65,68,72,73,74,75,76,77,78,82,83,84,85,86,97,115,116,119,120,121,122,123,124,125,126,127,133,134]. CP treatments significantly suppressed the data in the light blue boxes. The chemokines in blue were inhibited by CP treatments discriminately from OA treatments. Abbreviations: Th1, Th1 lymphocyte; Th2, Th2 lymphocyte; Th17, Th17 lymphocyte; E, eosinophils; M, macrophages; M1, macrophages type 1; M2a, macrophage M2a subtype.

**Figure 11:**
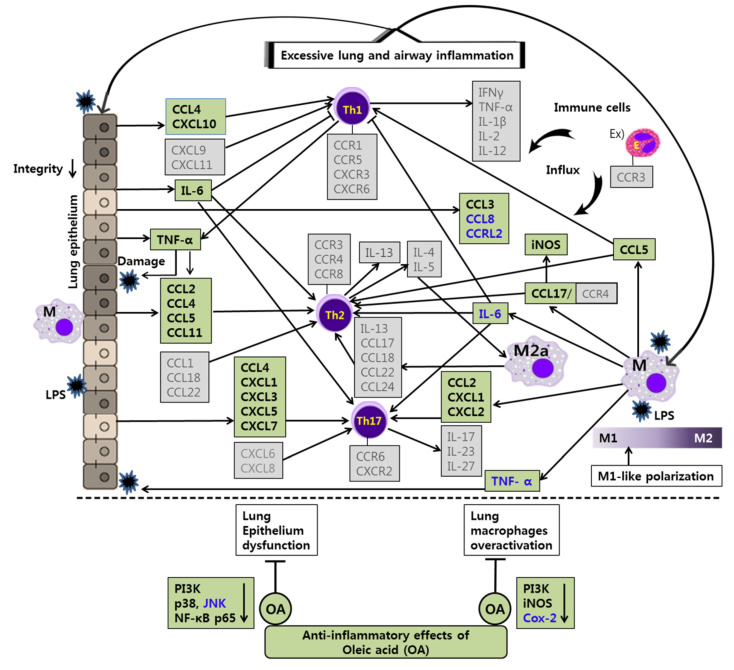
Schematic illustration of the anti-inflammatory effects and mechanisms of OA treatments in LPS-induced lung and airway inflammation in vitro. The lung epithelial cells and macrophages over-expressed extensive inflammatory chemokines and cytokines due to LPS stimulation, which leads to the recruitment of various immune cells and subsequently causes excessive lung and airway inflammation seen in asthmatics. The data in the gray boxes were cited from [16,17,18,20,21,32,33,39,50,51,52,53,55,58,59,63,64,65,68,72,73,74,75,76,77,78,82,83,84,85,86,97,115,116,119,120,121,122,123,124,125,126,127,130,133,134]. OA treatments significantly suppressed the data in the green boxes. The mediators in blue were inhibited by OA treatments discriminately from CP treatments. Abbreviations: Th1, Th1 lymphocyte; Th2, Th2 lymphocyte; Th17, Th17 lymphocyte; E, eosinophils; M, macrophages; M1, macrophages type 1; M2a, macrophage M2a subtype.

The anti-inflammatory effects and mechanisms of CP and OA treatments in lung epithelial cells and lung macrophages after LPS stimulation are illustrated in Figure 10 and Figure 11. Our integrated data offer a powerful approach that enables us to assess the full spectrum of chemokines in LPS-induced lung inflammation in vitro. These data provide new insights into lung and airway inflammation from the aspect of chemokines induced by lung epithelial cells and lung macrophages (Figure 10 and Figure 11). Our results show that CP and OA are potential chemokine-based therapeutic substances for treating lung and airway inflammation, and our findings could lay the groundwork for chemokine-based therapies in lung and airway inflammation if replicated in further animal models. We suggest that CP and OA can be utilized to apply chemokine-based anti-asthmatic therapies, given that our group’s previous study showed the efficacies of CP and OA in asthmatic mice [7].

## 5. Conclusions

We hypothesized that CP and OA treatments could demonstrate their anti-inflammatory effects by modulating inflammatory chemokines and their receptors in the lung and airway inflammation. Our study proves that CP and OA treatments exhibit anti-inflammatory effects on inflammatory chemokines and cytokines by inhibiting pro-inflammatory mediators, including PI3K, Akt, MAPKs, NF-κB, iNOS, and Cox-2, in LPS-induced lung inflammation in vitro. These findings suggest that CP and OA are potential chemokine-based therapeutic substances for treating the lung and airway inflammation seen in allergic disorders.

## Figures and Tables

**Table 1:** Primer sequences used in real-time PCR analysis.

Gene	Forward Primer	Reverse Primer
TNF-α	AATGGCCTCCCTCTCATCAGTT	CCACTTGGTGGTTTGCTACGA
IL-6	GAACAACGATGATGCACTTGC	TCCAGGTAGCTATGGTACTCC
CCL2	CAGCAGGTGTCCCAAAGAAG	TGTGGAAAAGGTAGTGGATGC
CCL3	GTACCATGACACTCTGCAACC	GTCAGGAAAATGACACCTGGC
CCL4	AACAACATGAAGCTCTGCGT	AGAAACACGAGGAAGTGGGA
CCL5	ATATGGCTCGGACACCACTC	TTCTTCGAGTGACAAACACG
CCL11	CCTGGACCAAAAACTCCAAA	GGCGACTGGTGCTGATATTC
CCL17	GGGATGCCATCGTGTTTCTG	GCCTTCTTCACATGTTTGTCTTTG
CXCL1	TGAGCTGCGCTGTCAGTGCCT	AGAAGCCAGCGTTCACCAGA
CXCL2	ATCCAGAGCTTGAGTGTGACG	GTTAGCCTTGCCTTTGTTCAG
CXCL5	GCATTTCTGTTGCTGTTCACGCTG	CCTCCTTCTGGTTTTTCAGTTTAGC
GAPDH	AACGGATTTGGCCGTATTGG	GCCTTGACTGTGCCGTTGAA

**Table 2:** Profile of 27 genes on the custom RT^2^ Profiler PCR array.

Ref. Seq	Gene Symbol	Gene Description
NM_016960	Ccl20	Chemokine (C-C motif) ligand 20
NM_009141	Cxcl5	Chemokine (C-X-C motif) ligand 5
NM_008176	Cxcl1	Chemokine (C-X-C motif) ligand 1
NM_011333	Ccl2	Chemokine (C-C motif) ligand 2
NM_013654	Ccl7	Chemokine (C-C motif) ligand 7
NM_021274	Cxcl10	Chemokine (C-X-C motif) ligand 10
NM_203320	Cxcl3	Chemokine (C-X-C motif) ligand 3
NM_011332	Ccl17	Chemokine (C-C motif) ligand 17
NM_019494	Cxcl11	Chemokine (C-X-C motif) ligand 11
NM_017466	Ccrl2	Chemokine (C-C motif) receptor-like 2
NM_021443	Ccl8	Chemokine (C-C motif) ligand 8
NM_001314054	Il6	Interleukin 6
NM_009140	Cxcl2	Chemokine (C-X-C motif) ligand 2
NM_013653	Ccl5	Chemokine (C-C motif) ligand 5
NM_023785	Ppbp	Pro-platelet basic protein
NM_011905	Tlr2	Toll-like receptor 2
NM_011338	Ccl9	Chemokine (C-C motif) ligand 9
NM_021704	Cxcl12	Chemokine (C-X-C motif) ligand 12
NM_008599	Cxcl9	Chemokine (C-X-C motif) ligand 9
NM_007721	Ccr10	Chemokine (C-C motif) receptor 10
NM_010045	Ackr1	Duffy blood group, chemokine receptor
NM_008337	Ifng	Interferon gamma
NM_009913	Ccr9	Chemokine (C-C motif) receptor 9
NM_013652	Ccl4	Chemokine (C-C motif) ligand 4
NM_007551	Cxcr5	Chemokine (C-X-C motif) receptor 5
NM_009139	Ccl6	Chemokine (C-C motif) ligand 6
NM_023158	Cxcl16	Chemokine (C-X-C motif) ligand 16

**Table 3:** Over-expressed or under-expressed genes in the LPS-stimulated vs. normal groups *.

Gene Symbol	Gene Description	Fold Regulation	*p*-Value
Ccl20	Chemokine (C-C motif) ligand 20	767.64	0.000000
Cxcl5	Chemokine (C-X-C motif) ligand 5	565.19	0.000001
Cxcl1	Chemokine (C-X-C motif) ligand 1	315.47	0.000000
Ccl2	Chemokine (C-C motif) ligand 2	109.80	0.000058
Cxcl10	Chemokine (C-X-C motif) ligand 10	70.39	0.000000
Cxcl11	Chemokine (C-X-C motif) ligand 11	58.68	0.000015
Ccl7	Chemokine (C-C motif) ligand 7	56.97	0.000000
Il6	Interleukin 6	56.47	0.000000
Cxcl3	Chemokine (C-X-C motif) ligand 3	25.54	0.000000
Ccl17	Chemokine (C-C motif) ligand 17	20.70	0.000004
Cxcl2	Chemokine (C-X-C motif) ligand 2	19.09	0.000015
Ccl8	Chemokine (C-C motif) ligand 8	18.35	0.000057
Ccrl2	Chemokine (C-C motif) receptor-like 2	14.22	0.000020
Tlr2	Toll-like receptor 2	9.57	0.000000
Ppbp	Pro-platelet basic protein	8.02	0.000142
Ccl4	Chemokine (C-C motif) ligand 4	7.48	0.000174
Ccl5	Chemokine (C-C motif) ligand 5	5.78	0.000001
Cxcl12	Chemokine (C-X-C motif) ligand 12	−4.42	0.000000
Ccl9	Chemokine (C-C motif) ligand 9	2.57	0.019087

* The fold-regulation threshold cut-off was set to 1.5. *p*-values were calculated based on a Student’s *t-*test of triplicate 2^(-Delta CT) values for each gene in the LPS-stimulated and normal groups. Only *p*-values less than 0.05 were included in the data.

**Table 4:** Over-expressed or under-expressed genes in the LPS-stimulated and CP 200 μg/mL-treated vs. LPS-stimulated groups *.

Gene Symbol	Gene Description	Fold Regulation	*p*-Value
Ccl4	Chemokine (C-C motif) ligand 4	−3.24	0.000553
Ccl9	Chemokine (C-C motif) ligand 9	−2.42	0.026609
Cxcl3	Chemokine (C-X-C motif) ligand 3	−2.18	0.000200
Ccl17	Chemokine (C-C motif) ligand 17	−1.98	0.000112
Cxcl2	Chemokine (C-X-C motif) ligand 2	−1.83	0.001428
Ccl6	Chemokine (C-C motif) ligand 6	−1.71	0.003535
Cxcl10	Chemokine (C-X-C motif) ligand 10	−1.65	0.000002
Ccl20	Chemokine (C-C motif) ligand 20	−1.57	0.000081
Ppbp	Pro-platelet basic protein	−1.57	0.015772

* The fold-regulation threshold cut-off was set to 1.5. *p*-values were calculated based on a Student’s *t-*test of triplicate 2^(-Delta CT) values for each gene in the LPS-stimulated plus CP 200 μg/mL-treated and LPS-stimulated groups. Only *p*-values less than 0.05 were included in the data.

**Table 5:** Over-expressed or under-expressed genes in the LPS-stimulated and CP 100 μg/mL-treated vs. LPS-stimulated groups *.

Gene Symbol	Gene Description	Fold Regulation	*p*-Value
Ccl4	Chemokine (C-C motif) ligand 4	−3.45	0.001698
Cxcl3	Chemokine (C-X-C motif) ligand 3	−2.13	0.000830
Ccl6	Chemokine (C-C motif) ligand 6	−1.95	0.026783
Ccl9	Chemokine (C-C motif) ligand 9	−1.94	0.040962
Ccl17	Chemokine (C-C motif) ligand 17	−1.81	0.000082
Ccl20	Chemokine (C-C motif) ligand 20	−1.64	0.000102
Cxcl2	Chemokine (C-X-C motif) ligand 2	−1.64	0.002071
Cxcl10	Chemokine (C-X-C motif) ligand 10	−1.57	0.000000

* The fold-regulation threshold cut-off was set to 1.5. *p*-values were calculated based on a Student’s *t-*test of triplicate 2^(-Delta CT) values for each gene in the LPS-stimulated plus CP 100 μg/mL-treated and LPS-stimulated groups. Only *p*-values less than 0.05 were included in the data.

**Table 6:** Over-expressed or under-expressed genes in the LPS-stimulated and OA 300 μM-treated vs. LPS-stimulated groups *.

Gene Symbol	Gene Description	Fold Regulation	*p*-Value
Ccl4	Chemokine (C-C motif) ligand 4	−6.49	0.000375
Cxcl10	Chemokine (C-X-C motif) ligand 10	−2.73	0.000002
Cxcl5	Chemokine (C-X-C motif) ligand 5	−2.27	0.000014
Ccl5	Chemokine (C-C motif) ligand 5	−1.85	0.000021
Cxcl3	Chemokine (C-X-C motif) ligand 3	−1.71	0.000763
Ccl8	Chemokine (C-C motif) ligand 8	−1.71	0.002909
Ccrl2	Chemokine (C-C motif) receptor-like 2	−1.65	0.001227
Ppbp	Pro-platelet basic protein	−1.53	0.020506

* The fold-regulation threshold cut-off was set to 1.5. *p*-values were calculated based on a Student’s *t-*test of triplicate 2^(-Delta CT) values for each gene in the LPS-stimulated plus OA 300 μM-treated and LPS-stimulated groups. Only *p*-values less than 0.05 were included in the data.

**Table 7:** Over-expressed or under-expressed genes in the LPS-stimulated and OA 100 μM-treated vs. LPS-stimulated groups *.

Gene Symbol	Gene Description	Fold Regulation	*p*-Value
Cxcl5	Chemokine (C-X-C motif) ligand 5	−3.20	0.000007
Ccl4	Chemokine (C-C motif) ligand 4	−2.33	0.001930
Cxcl3	Chemokine (C-X-C motif) ligand 3	−2.26	0.000248
Ppbp	Pro-platelet basic protein	−2.18	0.000922
Ccl8	Chemokine (C-C motif) ligand 8	−1.69	0.002448
Cxcl1	Chemokine (C-X-C motif) ligand 1	−1.59	0.000104

* The fold-regulation threshold cut-off was set to 1.5. *p*-values were calculated based on a Student’s *t-*test of triplicate 2^(-Delta CT) values for each gene in the LPS-stimulated plus OA 100 μM-treated and LPS-stimulated groups. Only *p*-values less than 0.05 were included in the data.

## Data Availability

All data are contained within the article or Supplementary Materials.

## Supplementary Materials

The following supporting information can be downloaded at: https://www.mdpi.com/article/10.3390/life12060857/s1. Table S1: Content of each fatty acid component detected in the ethanol extract of CP. Figure S1: The 17 fatty acids detected in CP ethanol extract by gas chromatography–flame ionization detection (GC-FID). (A) 17 out of 37 tested fatty acids were detected in CP ethanol extract. The data are expressed as g/100 g; refer to Table S1 for the common names of the lipids. (B) The top 10 fatty acids in order from top to bottom.
