# Supplementary material for: Anti-Inflammatory Effects of Cicadidae Periostracum Extract and Oleic Acid through Inhibiting Inflammatory Chemokines Using PCR Arrays in LPS-Induced Lung inflammation In Vitro
Source: Life (Basel). 2022 Jun 8;12(6):857. doi: 10.3390/life12060857 (PMC9225349; doi:10.3390/life12060857)
Supplement: Supplementary file 1 [file life-12-00857-s001.zip › life-1740030-supplementary.pdf]

Table S1. Content of each fatty acid component detected in the ethanol extract of CP. *

Lipid Numbers	Common Name	Results (g/100g)
C16:0	Palmitic acid	0.187
C18:1	Oleic acid	0.156
C18:0	Stearic acid	0.093
C18:2	Linoleic acid	0.057
C22:1n-9	Erucic acid	0.025
C14:0	Myristic acid	0.015
C16:1	Palmitoleic acid	0.015
C22:2	Docosadienoic acid	0.009
C20:1	Gondoic acid	0.008
C17:0	Heptadecanoic acid	0.006
C20:0	Arachidic acid	0.006
C15:0	Pentadecanoic acid	0.004
C22:0	Behenic acid	0.004
C24:0	Lignoceric acid	0.003
C6:0	Caproic acid	0.002
C8:0	Caprylic acid	0.002
C12:0	Lauric acid	0.002

*The data are expressed as g/100g

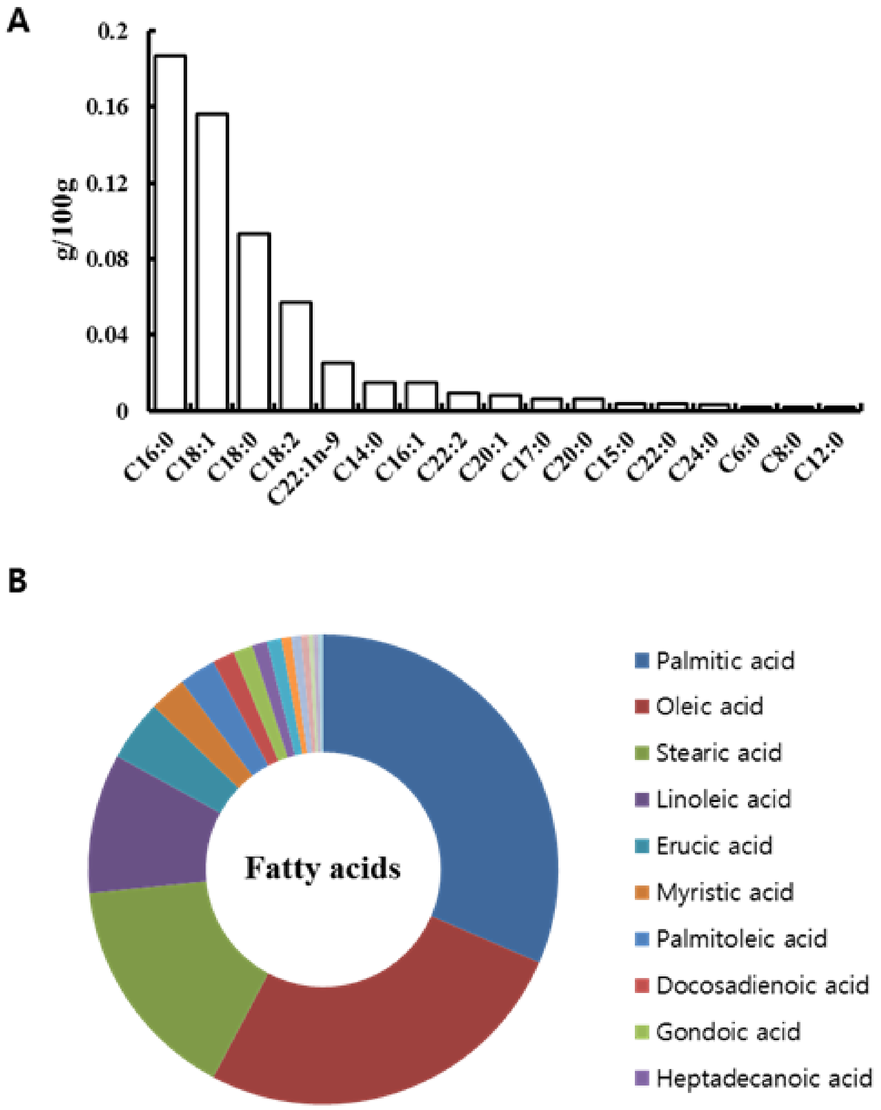

Figure S1. The 17 fatty acids detected in CP ethanol extract by gas chromatography-flame ionization detection (GC-FID). (A) 17 out of 37 tested fatty acids were detected in CP ethanol extract. The data are expressed as g/100g; refer to Table S1 for the common names of the lipids. (B) The top 10 fatty acids in order from top to bottom.
